# Activation of Si–H and B–H bonds by Lewis acidic transition metals and p-block elements: same, but different

**DOI:** 10.1039/d2sc02324e

**Published:** 2022-06-06

**Authors:** Pablo Ríos, Amor Rodríguez, Salvador Conejero

**Affiliations:** Instituto de Investigaciones Químicas (IIQ), Departamento de Química Inorgánica CSIC and Universidad de Sevilla, Centro de Innovación en Química Avanzada (ORFEO-CINQA) C/Américo Vespucio 49 41092 Sevilla Spain sconejero@iiq.csic.es marodriguez@iiq.csic.es

## Abstract

In this *Perspective* we discuss the ability of transition metal complexes to activate and cleave the Si–H and B–H bonds of hydrosilanes and hydroboranes (tri- and tetra-coordinated) in an electrophilic manner, avoiding the need for the metal centre to undergo two-electron processes (oxidative addition/reductive elimination). A formal polarization of E–H bonds (E = Si, B) upon their coordination to the metal centre to form σ-EH complexes (with coordination modes η^1^ or η^2^) favors this type of bond activation that can lead to reactivities involving the formation of transient silylium and borenium/boronium cations similar to those proposed in silylation and borylation processes catalysed by boron and aluminium Lewis acids. We compare the reactivity of transition metal complexes and boron/aluminium Lewis acids through a series of catalytic reactions in which pieces of evidence suggest mechanisms involving electrophilic reaction pathways.

## Abstract 2

In this *Perspective* we compare the ability of transition metals and p-block Lewis acids to activate electrophilically hydrosilanes and hydroboranes. The mechanistic similarities and dissimilarities in different catalytic transformations are analyzed.

## Graphical abstract

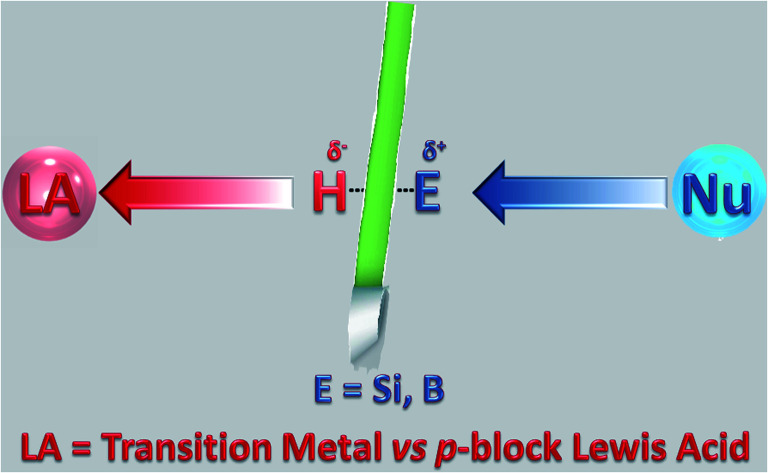

## 1. Introduction

The cleavage of silane and borane Si–H and B–H bonds by transition metal complexes is the first step for silylation and borylation of organic molecules.^1^ Usually, the bond breaking process involves an oxidative addition to the metal centre leading to silyl or boryl intermediates that then undergo reductive coupling with an organic fragment. In some cases, σ-bond metathesis or σ-CAM (Complex Assisted Metathesis) mechanisms can be operative while maintaining constant the oxidation state at the metal.^2^ However, there is more compelling evidence showing that an alternative reaction pathway can be involved in the heterolytic rupture of Si–H and B–H bonds, which generates highly reactive transient silylium or borenium/boronium-like cations together with metal hydrides.^3^ This type of reactivity is similar to that observed for some group 13 p-block Lewis acids (mainly boron and to a lesser extent aluminium), where oxidative addition steps are not possible. In other words, some transition metal complexes act as mere Lewis acids polarizing the E–H bond (E = Si, B) *via* formation of σ-EH complexes. This interaction considerably enhances the δ^+^ character at the E atom, conferring on it the chemical properties of silylium^4^ or borenium/boronium cations.^5^ Therefore, these systems can be engaged in new reactivity modes for the functionalisation of organic molecules, and in some cases they might possess enhanced functional group tolerance (something not always possible when other mechanisms are involved). The aims of this perspective article are (a) to compare the reactivity patterns and mechanistic features of transition metal complexes and boron/aluminium Lewis acids in a series of catalytic processes where electrophilic activation of the EH bonds is thought to take place, (b) to illustrate the possible similarities and dissimilarities from a mechanistic point of view and (c) to highlight the potential of transition metal complexes in the electrophilic activation of silanes^6^ and boranes.

## 2. Coordination modes of silanes and boranes

### 2.1 σ-SiH complexes

**Fig. 1:**
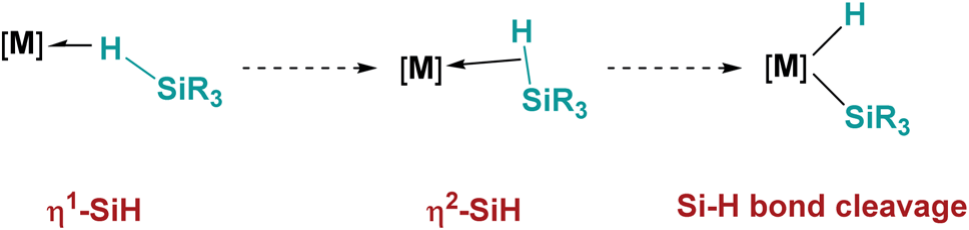
σ-SiH complexes and Si–H oxidative addition.

**Fig. 2:**
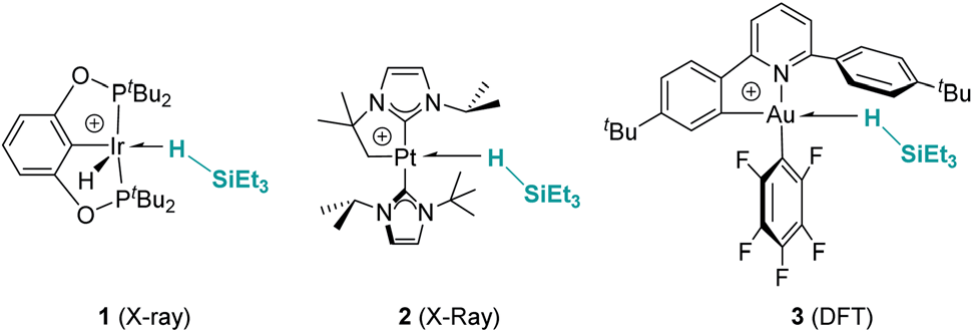
Examples of well-characterised σ-SiH transition metal complexes with the η^1^-coordination mode.

**Fig. 3:**
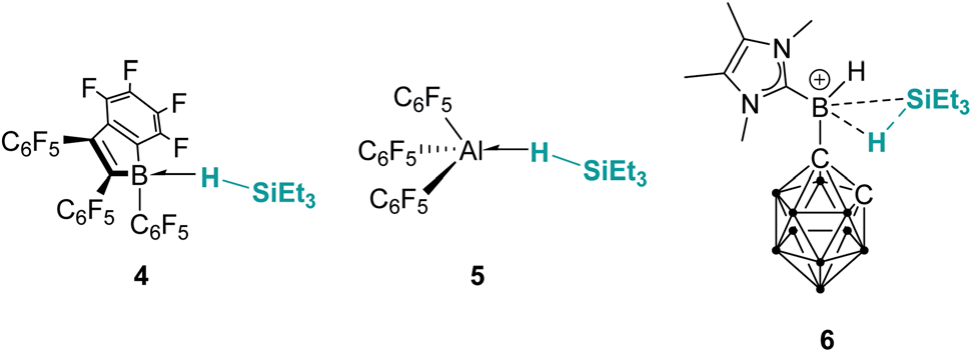
σ-SiH complexes of boron and aluminium.

Hydrosilanes have been known to form σ-SiH complexes with transition metals ever since Graham's seminal report on the interaction of dihydrosilanes with dinuclear rhenium carbonyl complexes.^7^ Since then, the number of well-defined complexes of this type has considerably increased and it is now well established that several bonding scenarios can be considered to explain the structural parameters observed.^8^ Excluding interligand or secondary interactions that are beyond the scope of this article,^9^ the binding of the silane with the metal can be viewed as a continuum on the way to cleave the Si–H bond (oxidative addition) through a transition from η^1^-SiH to η^2^-SiH binding modes (Fig. 1). The binding scenario will strongly depend on the ability of the metal centre to π-back-donate into the σ*_SiH_ orbital (whose energy depends in turn on the electronic properties of the substituents on the silane). It is therefore not surprising that most of the isolated σ-SiH complexes of transition metals exhibit an η^2^-SiH interaction. Nevertheless, if the metal centre is not sufficiently electron rich, η^1^-SiH (end-on) binding is possible. Still, steric effects can also account for imposing to some extent this bonding situation. This coordination mode is believed to increase the electrophilicity at the silicon atom,^3^ and is often involved in catalytic processes working through an ionic outer-sphere mechanism,^3*a*^ where a silylium ion can be transferred to a substrate with no redox events on the metal. There are only two reports in which this bonding situation has been observed crystallographically. In both cases the complexes are cationic in nature, which might diminish the ability of the metal to π-back-donate. The first example was reported by Brookhart *et al.* with the iridium complex [(POCOP)Ir(H)(HSiEt_3_)][B(C_6_F_5_)_4_] (POCOP = 2,6-[OP(^*t*^Bu)_2_]_2_C_6_H_3_) (Fig. 2, 1).^10^ Our group has recently isolated a new cationic example, namely [Pt(HSiEt_3_)(I^*t*^Bu^i^Pr′)(I^*t*^Bu^i^Pr)][BAr^F^] (I^*t*^Bu^i^Pr = 1-*tert*-butyl-3-isopropylimidazol-2-ylidene) (Fig. 2, 2).^11^ In both cases, DFT studies indicated that the degree of η^1^/η^2^ coordination can be controlled by the steric bulk of the ligands surrounding the metal centre. Thus, η^2^ coordination can be energetically accessible with smaller substituents.^12^ In fact, the platinum systems are thermally unstable and evolve through the cleavage of the Si–H bond, suggesting a very thin line between these two coordination modes.^12,13^ Recently, a σ-SiH complex of a gold(iii) cation (Fig. 2, 3) has been detected by spectroscopic techniques. DFT calculations suggest that this compound is better described as an η^1^-SiH derivative.^14^

**Fig. 4:**
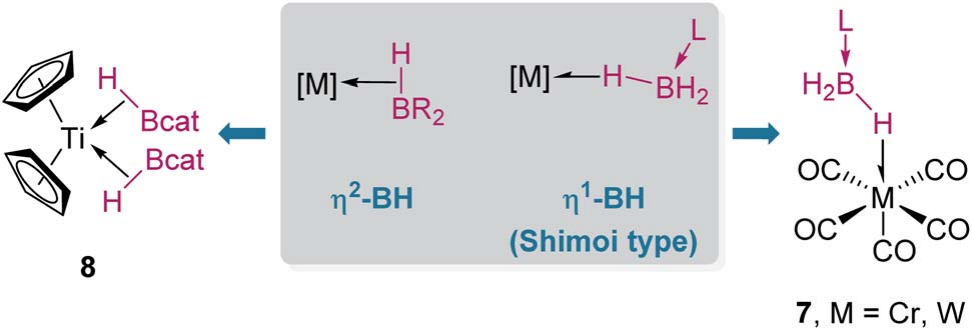
σ-BH complexes of tri- and tetracoordinated hydroboranes.

Main group elements are also known to form σ-SiH complexes, although very few examples have been structurally characterised. At variance with transition metals, the most common bonding mode of the silane is η^1^, since π-back-donation into the σ*_SiH_ orbital is either not possible or negligible. Tuononen, Piers and co-workers succeeded in isolating a borane–SiH complex (Fig. 3, 4).^15^ This type of interaction was previously proposed to account for the reactivity of electrophilic boranes such as B(C_6_F_5_)_3_ (ref. 16) that are known to electrophilically activate Si–H bonds to deliver silylium cations. In their study the authors showed that the most stable coordination mode was of η^1^ type.

**Fig. 5:**
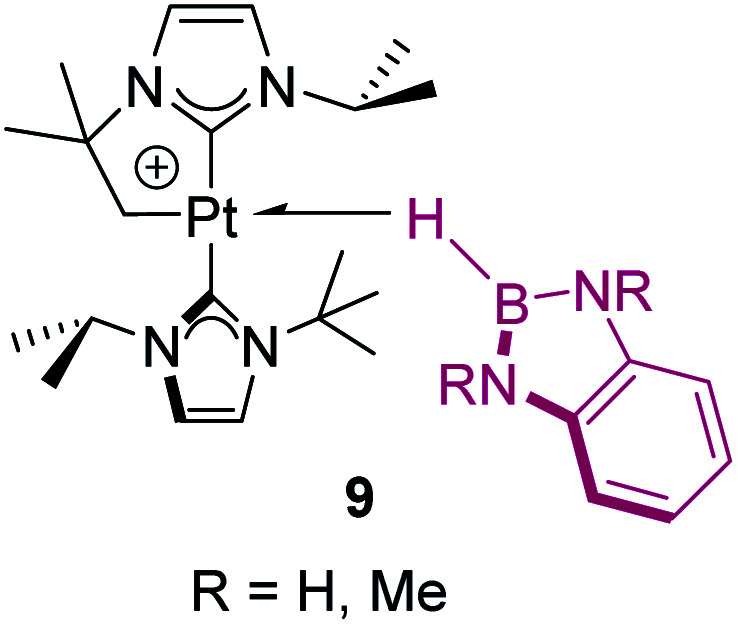
Platinum σ-BH complexes with an η^1^ coordination mode.

Similarly, Chen and co-workers described the isolation of the first σ-SiH complex of an electrophilic aluminium system (Fig. 3, 5), again displaying the end-on coordination of the silane.^17^ However, Wang, Li and co-workers have isolated a derivative in which the interaction of Et_3_SiH with a borenium ion is, according to the authors, better described as η^2^ (compound 6 in Fig. 3), as a result of the strong interaction of the σ-Si–H bond with the empty p orbital of boron and the low steric constraints exerted by the hydrogen-substituted borenium cation.^18^ Still, the silicon atom of this compound exhibits a highly electrophilic character and therefore is able to transfer a silylium ion to nucleophiles.

### 2.2 σ-BH complexes

When discussing σ-BH complexes a distinction between those formed by tri-(R_2_BH) and tetra-coordinated boranes (L·BH_3_, L = Lewis base) should be made to understand the coordination modes prevalent for each type of borane (Fig. 4).^19^ As in σ-SiH complexes the bonding description to a metal centre has two main contributions: σ donation from the σ-B–H bond and π-back donation to either the σ*_BH_ orbital or the p_*z*_ orbital of boron. The σ*_BH_ orbital is usually rather high in energy for tetra-coordinated boranes, and therefore π-back donation to this orbital is not relevant. Since the p_*z*_ orbital is filled by the interaction with the Lewis base, the most common coordination mode for these systems is η^1^-BH (usually named Shimoi type coordination; Fig. 4, 7).^20^

In contrast, tri-coordinated boranes have an available p_*z*_ orbital, thus the predominant coordination mode is η^2^-BH, as determined for example in the seminal work by Hartwig *et al.* (Fig. 4, 8).^21^ Nevertheless, we have isolated a platinum σ-BH complex (Fig. 5, 9) of a tri-coordinated borane where the binding mode is better described (according to DFT calculations) as η^1^-BH, thus sharing some structural similarities with complex 2 (Fig. 2).^22^ In any case, the σ-BH complexes can be considered to some extent as arrested intermediates leading to B–H bond cleavage as for silanes (Fig. 1), by means of oxidative addition processes. However, some of the Shimoi type complexes, particularly those of cationic nature, have been described to undergo heterolytic B–H bond splitting leading to metal hydrides and boronium cations [BH_2_(L)_2_]^+^ due to the high degree of polarization of this bond.^23^

Interestingly, we are not aware of the isolation of σ-BH compounds (with either tri- or tetra-coordinated boranes) possessing boron or aluminium-based Lewis acids of similar nature to those reported with silanes, although some of them have been suggested as intermediates (by DFT methods) in catalytic reactions.^24^

## 3. Catalytic silylation reactions

### 3.1 Reduction of carbonyl substrates

#### 3.1.1 Reduction of carbonyl substrates by main group Lewis acids

**Scheme 1:**
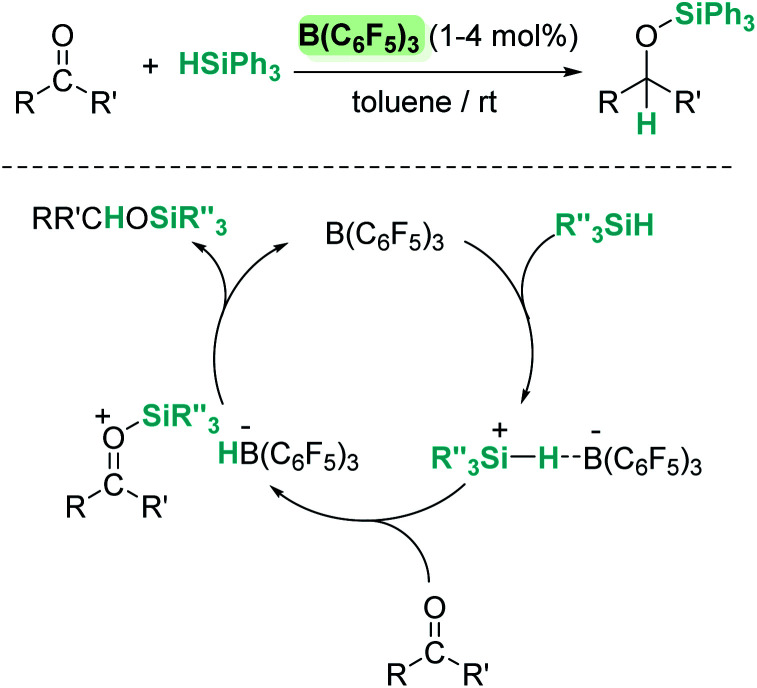
Hydrosilylation of carbonyl compounds by B(C_6_F_5_)_3_ and mechanistic proposal.

More than 20 years ago the catalytic competence of B(C_6_F_5_)_3_ in the hydrosilylation of carbonyl functional groups was established by Piers and coworkers.^16^ B(C_6_F_5_)_3_ proved to be an excellent catalyst for the high yielding (75–96%) reduction of aromatic and aliphatic aldehydes and ketones using Ph_3_SiH as a reductant (Scheme 1). A rigorous control of the stoichiometry was required to avoid over-reduction of products. Esters were reduced as well, but over-reduction of the initial silylacetal species could not be eluded, with formation of hydrocarbons and bis(silyl)ethers in different ratios.^16^

In contrast to what was believed at that time, Piers demonstrated that B(C_6_F_5_)_3_/carbonyl adducts are not implicated in the addition of the Si–H bond across the C–O bond. Based on experimental observations they suggested that the key step is the electrophilic silane activation by B(C_6_F_5_)_3_ to form a σ-silane borane complex [(C_6_F_5_)_3_B⋯HSiR_3_], analogous to that described in the Introduction section.^15^ This compound reacts with the carbonyl species to generate a silylium cation having a borohydride counteranion [RR′COSiR_3_][HB(C_6_F_5_)_3_] (Scheme 1). Hydride delivery to the carbonyl carbon atom releases the silylated product, closing the catalytic cycle.^25^ Analysis of the stereochemistry of this reaction, using a silicon stereogenic silane, confirmed the inversion of the silane configuration. This is in agreement with a concerted S_N_2-type mechanism for the displacement of the borohydride anion (HB(C_6_F_5_)_3_^−^) by attack of the silicon atom by the carbonyl substrate (Scheme 1). The existence of free silylium ions was ruled out based on experimental evidence using a deuterium-enriched silane, excluding an S_N_1-type mechanism.^26^

**Scheme 2:**
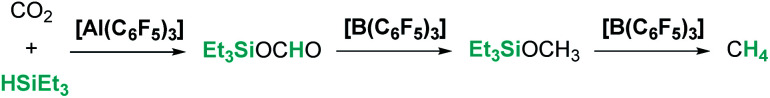
CO_2_ reduction by Et_3_SiH using a combination of Al(C_6_F_5_)_3_ and B(C_6_F_5_)_3_.

The aluminium species Al(C_6_F_5_)_3_ is considered a stronger Lewis acid than the boron analogue and it has also been tested in the catalytic hydrosilylation of ketones (2 mol% catalyst loading, 16–95% yield). This species exhibits much lower activity than B(C_6_F_5_)_3_ since its higher oxophilicity and Lewis acidity allow the formation of more stable aluminium–ketone adducts [R_2_CO⋯Al(C_6_F_5_)_3_], thereby reducing the amount of free aluminium available for the electrophilic silane activation.^17^ Interestingly, the combination of both systems, B(C_6_F_5_)_3_ and Al(C_6_F_5_)_3_, revealed some features that make them complementary catalysts for carbon dioxide reduction to methane.^27^ Chen and co-workers showed that, in the absence of B(C_6_F_5_)_3_, Al(C_6_F_5_)_3_ allows the effective reduction of carbon dioxide to form a stable aluminium-silyl formate species that does not undergo further hydrosilylation. However, when a mixture of both Lewis acids is used, further reduction to bis(silyl)acetal, methyl silyl ether and methane is observed (the highest yield of methane (94%) was obtained using 5 mol% B(C_6_F_5_)_3_/1 mol% Al(C_6_F_5_)_3_ after 30 hours at 80 °C; Scheme 2). Extensive mechanistic analysis demonstrated that the weaker substrate interaction between the hydrosilylated products and B(C_6_F_5_)_3_ (compared to Al(C_6_F_5_)_3_) enables the electrophilic activation of silane, making catalytic turnover possible. It is worth mentioning that Al(C_6_F_5_)_3_ is able to reduce CO_2_ to methane but with a very low activity (10 mol%, 80 °C, 24 h, 16% CH_4_), whereas B(C_6_F_5_)_3_ is totally ineffective. Interestingly, carbon dioxide can be fully reduced to methane (10 mol%, 80 °C, 60 h, 70% yield) using cationic aluminium species [AlEt_2_][CH_6_B_11_I_6_] and related derivatives.^28^

Bergman *et al.* reported the effective hydrosilylation of a variety of aldehydes, ketones and imines by the four-coordinate cationic aluminium alkyl complex supported by scorpionate ligands [Tp*AlMe][MeB(C_6_F_5_)_3_] (Tp* = hydro-(1,3-dimethylpyrazol-1-yl)borate) (1–2.5 mol% catalyst loading, 70–100 °C, 0.5–1 h, 70–99% yield). Although no detailed analysis of the mechanism of this reaction is provided, experimental results support the formation of an aluminium–silane adduct similar to that suggested in reactions catalysed by B(C_6_F_5_)_3_.^29^

#### 3.1.2 Reduction of carbonyl substrates by a combination of main group Lewis acids and transition metal complexes

**Scheme 3:**
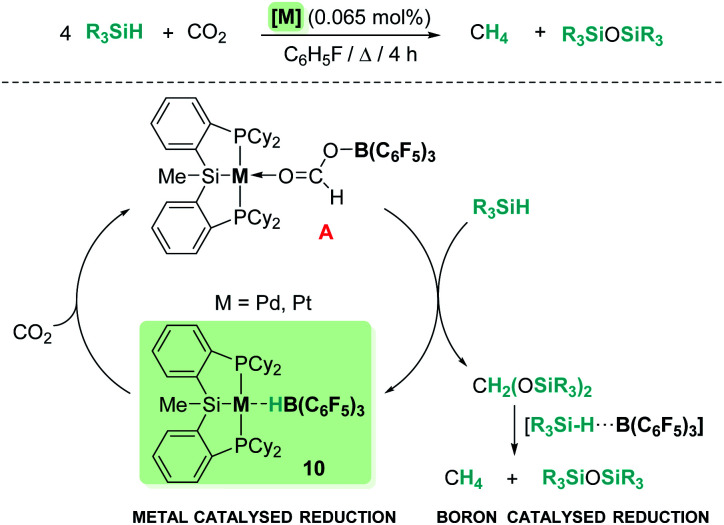
Hydrosilylation of CO_2_ catalysed by complexes [(Cy-PSiP)M][HB(C_6_F_5_)_3_], 10.

One of the main challenges in main group catalysis is reaction control over the ratio of the resulting products. However, modification of the ligands to increase the steric hindrance around the main group catalyst usually compromises its catalytic activity. An interesting strategy to improve the catalytic performance is the design of mixed catalysts by combination of a transition metal complex and a main group Lewis acid, with the former involved in the initial reduction of CO_2_ and the latter being responsible for the activation of the Si–H bond. In 2012, Turculet *et al.* published the highly selective hydrosilylation of carbon dioxide to methane catalysed by complex [(Cy-PSiP)M][HB(C_6_F_5_)_3_] (Scheme 3, 10) (prepared from pincer (η^2^-SiH) complexes [(Cy-PSiHP)M] (M = Pd and Pt) and B(C_6_F_5_)_3_) (Pd: 85 °C, TON 465; Pt: 65 °C, TON 1063).^30^ The mechanism of this reaction involves initial reduction of carbon dioxide with formation of a formaborate metal complex [(Cy-PSiP)M][O_2_CHB(C_6_F_5_)_3_] (A, Scheme 3) which, upon reaction with two equivalents of silane, releases bis(silyl)acetal and regenerates the catalyst. Then, two subsequent metal-free reduction steps catalysed by B(C_6_F_5_)_3_ yield methyl silyl ether and methane through electrophilic activation of two additional molecules of silane.

**Scheme 4:**
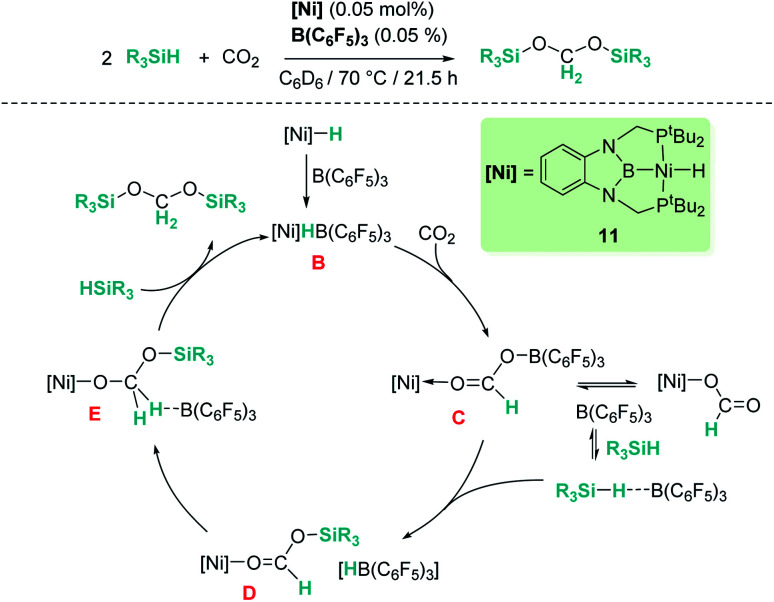
Hydrosilylation of CO_2_ catalysed by complex [(^*t*^Bu-PBP)NiH], 11.

Our group reported a similar catalytic system based on a pincer nickel hydride complex [(^*t*^Bu-PBP)NiH] (11, Scheme 4) that selectively reduces carbon dioxide to bis(silyl)acetal (60% yield) in the presence of B(C_6_F_5_)_3_.^31^ Similarly, CO_2_ activation by insertion into the Ni–H bond of the ion pair [(^*t*^Bu-PBP)Ni][HB(C_6_F_5_)_3_] (B) constitutes the first step of the catalytic reaction. Then, the rate limiting step is B(C_6_F_5_)_3_ dissociation from the active species [(PBP)Ni(OCHOB(C_5_F_6_)_3_)] (C), which controls the amount of free borane that can lead to over-reduction to methane. Free borane activates the silane by formation of [R_3_Si–H⋯B(C_6_F_5_)_3_], then the silylium cation (R_3_Si^+^) is transferred to the oxygen atom of the nickel formate to form [(PBP)NiOCHOSiMe_3_][HB(C_6_F_5_)_3_] species D. Hydride transfer yields intermediate [(PBP)NiOCH_2_OSiMe_3_] (E) and B(C_6_F_5_)_3_ that activates a new molecule of silane. In the last step, reaction of E with the newly formed silylium cation releases bis(silyl)acetal with regeneration of the ion pair [(PBP)Ni][HB(C_6_F_5_)_3_]. Here, no over-reduction products were formed due to the effective sequestration of free borane by nickel species C and B, according to DFT calculations.^31*b*^

**Fig. 6:**
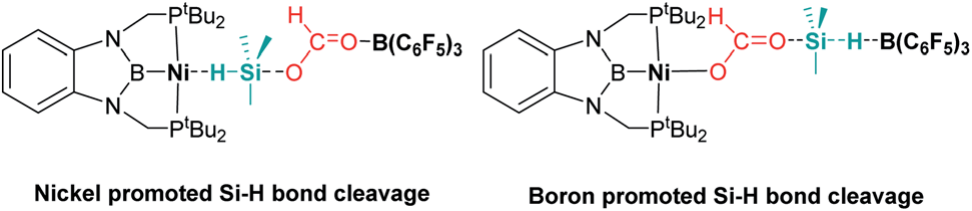
Alternative intermediate proposed by Ke *et al.* for the hydrosilylation of CO_2_ catalysed by complex 11 and B(C_6_F_5_)_3_.

Ke and co-workers proposed an alternative mechanism that contemplates nickel promoted Si–H bond activation (Fig. 6, left) as the preferred pathway, avoiding the B(C_6_F_5_)_3_ dissociation step. They found very similar energy barriers for both pathways, with the heterolytic rupture of the Si–H bond being the rate-determining step in this case.^32^

**Scheme 5:**
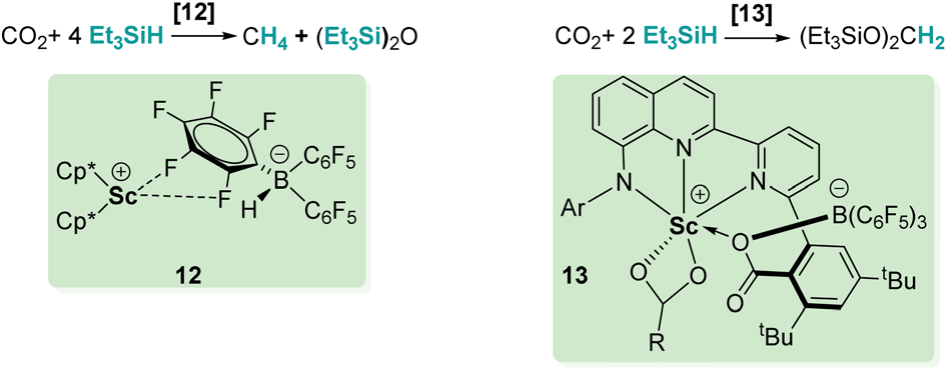
Hydrosilylation of CO_2_ by mixed Sc/B systems.

Different combinations of metallic species (zirconium, scandium, rhenium) and B(C_6_F_5_)_3_ have been tested for the selective hydrosilylation of carbon dioxide.^33^ Piers and co-workers demonstrated that the design of the tandem metal/borane system is determinant to minimize the amount of free borane present in solution to allow a rational selectivity control.^33*c*^ They observed that when using [Cp*_2_Sc][HB(C_6_F_5_)_3_] (12, Scheme 5), the catalyst is incapable of trapping the free borane formed during the reaction and reduction products are rapidly transformed into methane. However, substitution of Cp* fragments by an anilido bipyridyl ligand (complex 13, Scheme 5) efficiently regulates the amount of free borane present in the catalytic media and allows an exquisite control of the selectivity to exclusively form bis(silyl)acetal.^33*a*^

From these examples, it can be extracted that the incorporation of metal complexes into systems solely constituted by main group elements seems to have a beneficial effect on the selectivity control of the reaction, which can also be tuned *via* ligand design around the transition metals.

#### 3.1.3 Reduction of carbonyl substrates by transition metal complexes

**Scheme 6:**
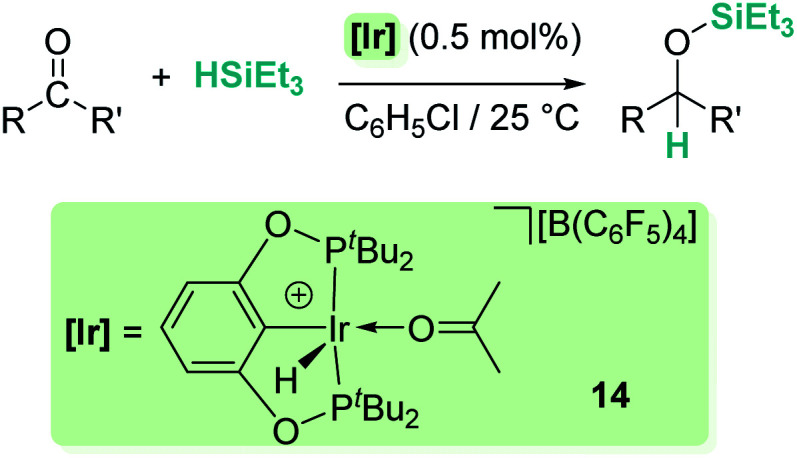
Hydrosilylation of carbonyl compounds catalysed by 14.

**Scheme 7:**
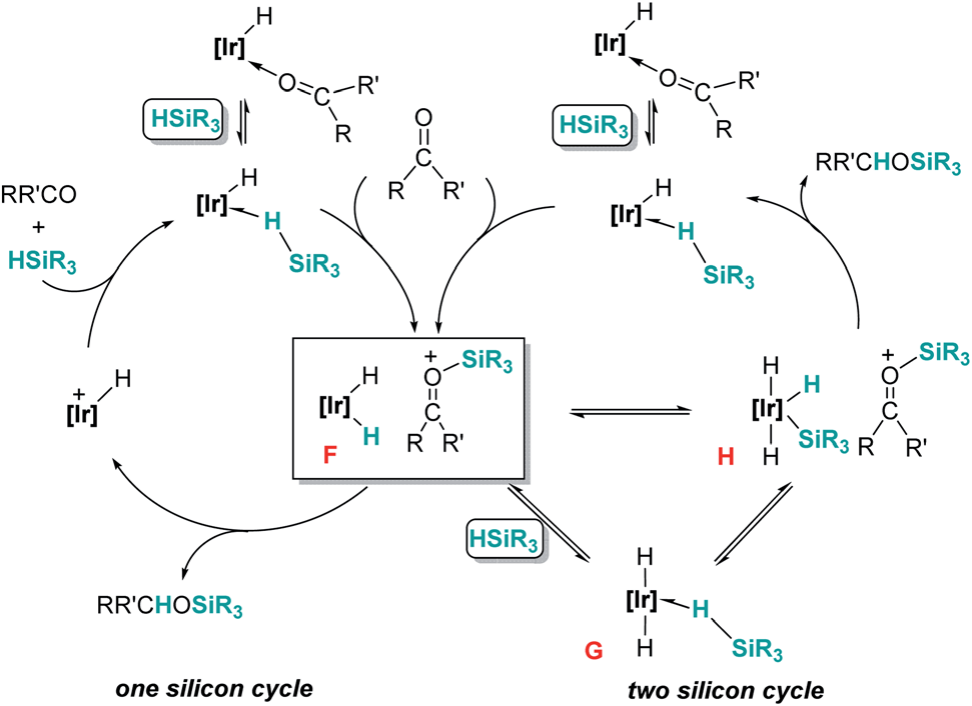
Mechanistic initial (left) and modified (right) proposals for the hydrosilylation of ketones by complex 14.

**Scheme 8:**
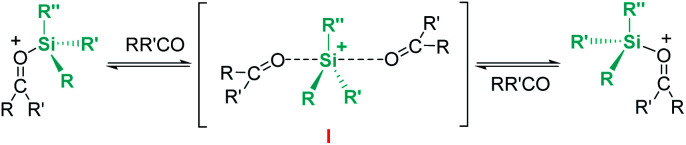
Racemisation of silicon-stereogenic silanes.

Related to systems exclusively based on transition metals, one of the earliest representative examples of the ionic outer-sphere mechanism was reported by Brookhart using the iridium complex [(POCOP)IrH(acetone)][B(C_6_F_5_)_4_] (14, Scheme 6).^34^ This system proved to be an active catalyst for the hydrosilylation of a great variety of carbonyl substrates including ketones, aldehydes, ethers, amides, esters and carbon dioxide. The hydrosilylation of ketones and aldehydes to obtain silyl ethers was successfully accomplished with excellent yields (quantitative conversion in 20–30 min).^34*a*^ Both alkyl and aryl ketones with very bulky substituents were reduced. For aryl ketones higher activities were observed for the more basic ones. Precise control of the amount of silane allows the selective reduction of aldehydes to silyl ethers but over-reduction products were detected when an excess of silane was used. The reduction of esters and amides is not selective and over-reduction products were obtained. Based on previous studies with alkyl halides and alkyl ethers,^34*b*,*c*^ the authors originally proposed a similar mechanism for the hydrosilylation of ketones. This consists of the electrophilic activation of silane by the cationic iridium complex and S_N_2 nucleophilic attack of the ketone, to yield a siloxy-carbenium ion pair with an iridium dihydride complex (F, Scheme 7). In a final step, hydride transfer to the siloxycarbenium ion releases the silyl ether product (Scheme 7, left). However, in 2014 Oestreich *et al.* reported an extensive analysis of this mechanism and provided new experimental evidence of a key step that involves the participation of an additional silane molecule (two silicon cycles shown in Scheme 7, right).^35^ It was demonstrated that the iridium dihydride complex F was not able to reduce the siloxy-carbenium. Instead, two new species (either an iridium(iii) dihydride–silane adduct G or an iridium(v) trihydride silyl complex H) can facilitate the hydride transfer to finally release the silyl ether. Additionally, it was found that when silicon-stereogenic silanes are used, racemisation of the final product is observed at low silane concentrations. This observation raises questions about an S_N_2-type nucleophilic addition pathway previously proposed, where Walden inversion is expected. The authors suggested that the siloxy-carbenium ion is in equilibrium with a new species (I in Scheme 8), formed by the reversible coordination of a second molecule of ketone to the silicon atom. This process, which is in competition with the hydride transfer step, is responsible for the racemisation event (Scheme 8).

Based on the knowledge extracted from this system, the possibility of a similar mechanism involving two molecules of silane for the hydrosilylation of ketones and aldehydes catalysed by the ruthenium complex [Cp(PPh_3_)Ru(NCMe)_2_][B(C_6_F_5_)_4_] was also investigated by Oestreich and co-workers.^36^ The authors came to the same conclusions; in this particular case, the hydride transfer event is promoted by the neutral ruthenium(iv) dihydride silyl complex [Cp(PPh_3_)Ru(H)_2_(SiR_3_)].

**Scheme 9:**
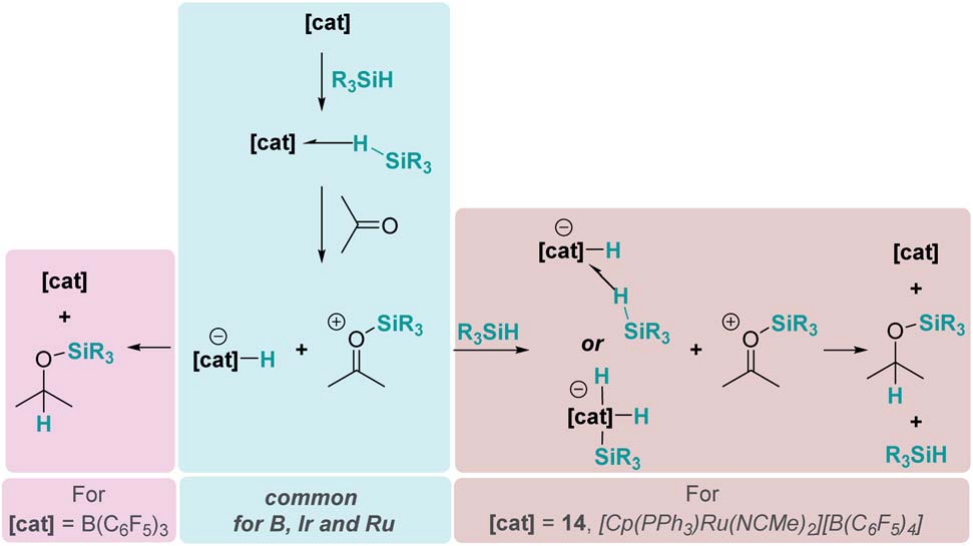
Reaction pathways for ketone hydrosilylation by boron, iridium and ruthenium complexes.

The examples mentioned above provide an excellent opportunity to comment on the differences and similarities between the mechanisms for the hydrosilylation of ketones by electrophilic activation of silanes promoted by main group Lewis acids (boron or aluminium) and transition metals. The bonding situation in the activation of silanes by transition metals resembles that encountered with main group Lewis acids, with the metal playing the role of the main group element (Scheme 9). In both cases, the heterolytic cleavage of the activated Si–H bond by nucleophilic attack by the carbonyl substrate generates a silylium cation and a hydride species (borohydride *vs.* transition metal hydride complex). From this point, the hydride transfer follows different pathways depending on the system. The hydride transfer seems to be directly from the borohydride complex, but the hydridic character of the metal complex needs to be enhanced by the assistance of another molecule of silane, thus two equivalents of silane are required in each catalytic cycle.

There is an interesting analysis by Wei *et al.* comparing Brookhart's catalyst [(POCOP)IrH(acetone)][B(C_6_F_5_)_4_], 14, and the analogous iron complex [(POCOP)FeH(PMe_3_)_3_] reported by Guan.^37^ DFT analysis of the hydrosilylation mechanism of carbonyl compounds revealed significant differences between both systems. While the ionic outer-sphere pathway is favoured for the iridium complex, the iron system seems to operate through a different mechanism in which the initial coordination of the carbonyl substrate (η^1^- or η^2^-adduct) is proposed. Thereafter, migratory insertion of the carbonyl substrate into the Fe–H bond yields an iron alkoxide species, followed by σ-bond metathesis between the Si–H bond and the metal alkoxide bond, releasing the silyl ether product. The authors claimed that the reason behind the different behaviours of the two catalysts is the difference in the M–H bond strength (Ir–H *vs.* Fe–H). The higher Ir–H bond strength precludes migratory insertion of the carbonyl compound into the Ir–H bond, favouring the ionic pathway. In contrast, the energy barrier for this step is considerably lower in the case of the iron complex, favouring the carbonyl pre-coordination pathway.

**Scheme 10:**
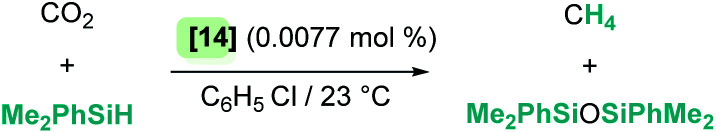
Hydrosilylation of CO_2_ to methane catalysed by 14.

Hydrosilylation of more challenging substrates such as carbon dioxide was also reported by Brookhart *et al.*^38^ The electrophilic iridium η^1^-silane complex 1 (generated from complex 14) reacts with CO_2_ to yield silyl formate, which is rapidly reduced to bis(silyl)acetal and methyl silyl ether, and ultimately methane (Scheme 10). The selectivity of this reaction strongly depends on the steric bulk of the silane. Reduction of the C–O bond whether to bis(silyl)acetal (R_3_SiO)_2_CH_2_ or silyl ether (R_3_SiOMe) species becomes more difficult when bulky silanes are used, giving rise to a mixture of products. However, methane is the only reaction product if the silane is smaller (quantitative conversion).

**Scheme 11:**
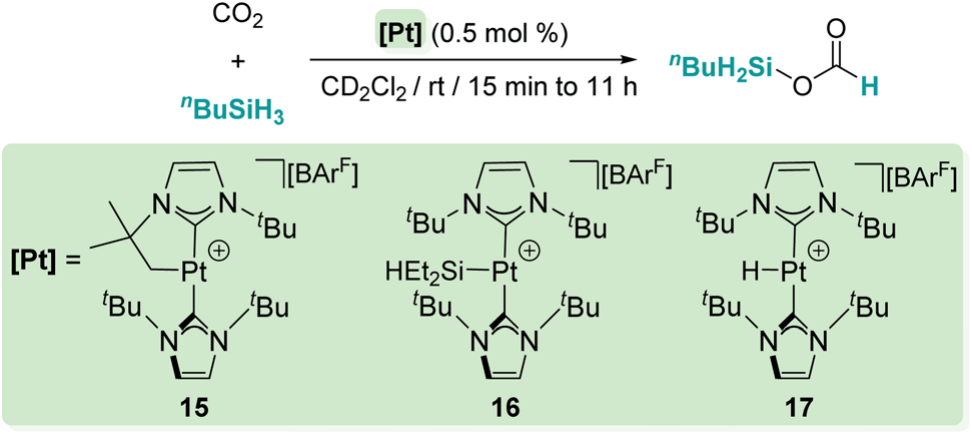
Selective hydrosilylation of CO_2_ catalysed by complexes 15–17.

Our group has also reported that the cationic 14-electron platinum complexes [Pt(I^*t*^Bu′)(I^*t*^Bu)][BAr^F^] (15), [Pt(SiEt_2_H)(I^*t*^Bu)_2_][BAr^F^] (16) and [Pt(H)(I^*t*^Bu)_2_][BAr^F^] (17) catalyse the selective reduction of CO_2_ to silyl formate derivatives with primary or secondary silanes at room temperature, without observing further over-reduction products (Scheme 11).^39^

**Scheme 12:**
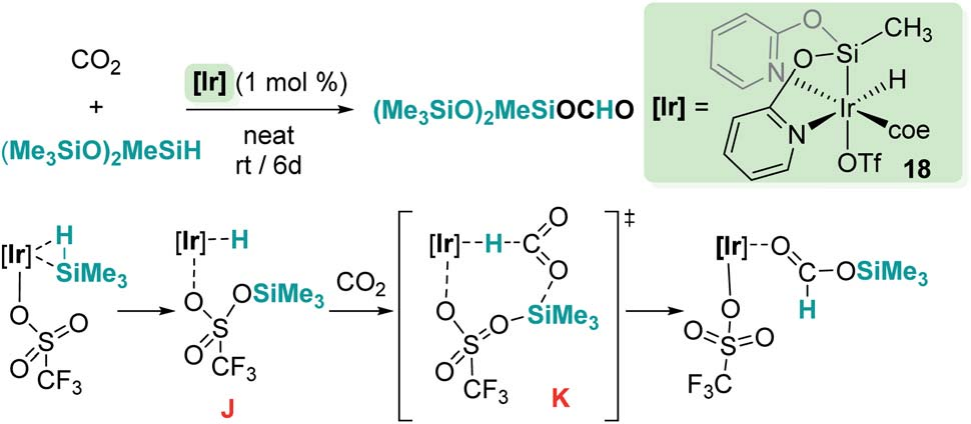
Hydrosilylation of CO_2_ by complex 18 and mechanistic proposal.

Reduction of CO_2_ to silyl formate was also described by Oro, Fernández-Álvarez and co-workers using the iridium system [Ir(H)(coe)(CF_3_SO_3_)(NSiN)] (18) (NSiN: bis(pyridine-2-yloxy)methylsilyl; coe: cyclooctene) and HSiMe(OSiMe_3_)_2_ as a reductant (Scheme 12).^40^ At variance with Brookhart's system, the electrophilic activation of the Si–H bond proceeds through a cooperative mechanism that involves the trifluoromethanesulfonate (OTf) ligand and the iridium centre. Then, after the heterolytic rupture of the Si–H bond, a hydride is transferred to the metal and a silylium cation binds one of the oxygen atoms of the OTf ligand (intermediate J in Scheme 12). From this point, CO_2_ reduction to silyl formate occurs through a concerted pathway (transition state K) in which both hydride and silyl groups are transferred to the carbon and oxygen atoms of carbon dioxide, respectively.

**Scheme 13:**
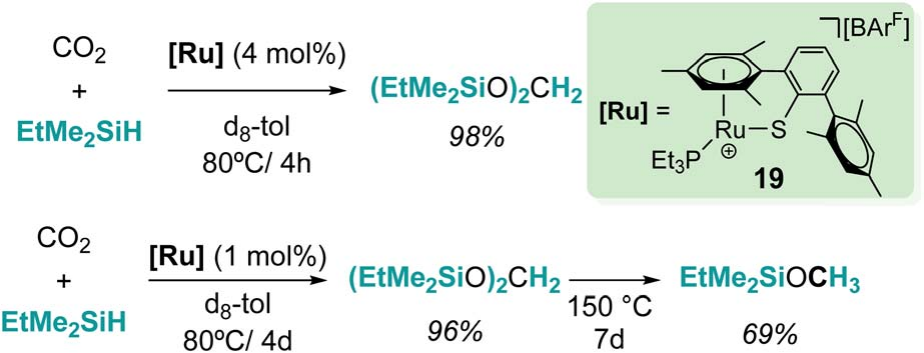
Hydrosilylation of CO_2_ catalysed by complex 19.

The cationic coordinatively unsaturated ruthenium thiolate complex [(R_3_P)–Ru(SDmp)][BArF_4_] (19, Scheme 13) proved to be an active catalyst for the reduction of CO_2_ to bis(silyl)acetal or methyl silyl ether depending on the reaction temperature, the latter being favoured at higher temperatures. Similarly, the authors proposed that a ruthenium–thiolate cooperative mechanism allows the electrophilic activation of the Si–H bond, generating a ruthenium hydride and a sulfur-stabilized silylium cation.^41^

**Scheme 14:**
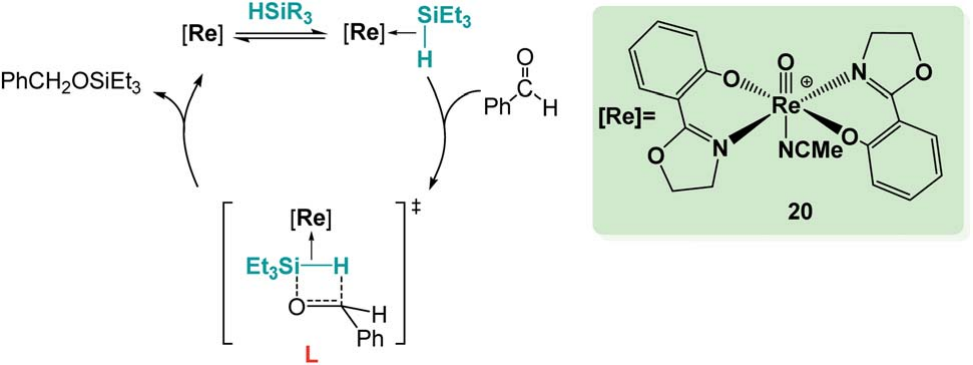
Mechanistic proposal for hydrosilylation of ketones mediated by complex 20.

Sometimes, the reaction mechanisms might be more complex than expected and alternative reaction pathways have been described. Abu-Omar *et al.* reported that complex [Re(O)(hoz)_2_][B(C_6_F_5_)_4_] (20, Scheme 14) was very efficient for ketone hydrosilylation. The reaction proceeds in the absence of solvent under atmospheric conditions, yielding the corresponding silyl ethers in good yields (0.1 mol%, 71–86%).^42^ Analysis of the mechanism of this reaction offered experimental evidence for an alternative pathway, in which the initial formation of an η^2^-silane rhenium species (or η^1^, according to DFT calculations by Wei)^42*c*^ is proposed. After electrophilic activation of the silane by the metal centre, a concerted mechanism where the hydride is transferred to the carbonyl group activated by silicon is suggested (transition state L in Scheme 14). The authors showed that the addition of the silane across the Re–O bond does not occur in the catalytic hydrosilylation of carbonyl substrates and claimed that the low hydridic character of the [(hoz)_2_Re(O)H] species precludes a mechanism involving the insertion of the carbonyl group into the Re–H bond.^42*a*,*c*^

In some occasions, determining the mechanism for hydrosilylation of carbonyl compounds can be challenging. This is the case when complex MoCl_2_O_2_ is used, for which several proposals have been described.^43^ However, a subsequent computed analysis performed by Wei *et al.* disclosed an ionic outer-sphere pathway as the most favourable route.^44^ The authors argued that the high Lewis acidity of the molybdenum centre favours the initial formation of an η^1^-silane molybdenum adduct. Then, nucleophilic attack of the carbonyl substrate provokes the heterolytic cleavage of the Si–H bond to give the final product after hydride transfer from the metal.

**Scheme 15:**
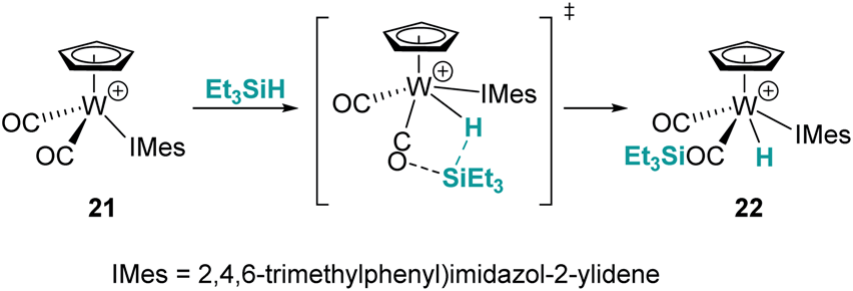
Mechanistic proposal for the activation of Si–H bonds by 21.

Likewise, different mechanistic proposals have been reported for the cationic tungsten(ii) complex [CpW(CO)_2_(IMes)][B(C_6_F_5_)_4_] (21) originally reported by Bullock *et al.*^45^ Initially, the first step of the mechanism for the hydrosilylation of ketones was described to proceed *via* oxidative addition of the silane to yield a tungsten(iv) complex but, later on, Wei *et al.* published a different proposal suggesting the intermediacy of an η^1^-silane complex.^46^ Further combined experimental and theoretical analysis by Oestreich *et al.* supports a cooperative mechanism in which a CO ligand participates in the Si–H bond activation step, giving rise to the formation of a tungsten hydride complex bearing a carbonyl-stabilised silylium cation, 22 (Scheme 15).^47^ This complex was used for the solvent-free hydrosilylation of ketones with good rates, high conversions and excellent selectivity. The formation of a liquid clathrate that contains a few molecules of substrate per molecule of the catalyst was decisive for the high catalytic performance and recycling of the catalytic species (using catalyst loadings of 0.2 mol%). The molybdenum derivative was also tested but showed lower catalytic activity compared to the tungsten complex.

The examples mentioned above serve as proof of the complexity associated with the mechanistic understanding of electrophilic activation of silanes by transition metal complexes, making evident that a generally accepted mechanism may be reconsidered to find a similar or even more energetically favourable alternative pathway. In addition, at variance with main group catalysts, the mechanism for the electrophilic activation of Si–H might involve the cooperative participation of the ligands around the metal center.

### 3.2 Dehydrocoupling processes of alcohols and silanes leading to Si–O bonds

#### 3.2.1 Dehydrocoupling processes mediated by transition metals

**Scheme 16:**
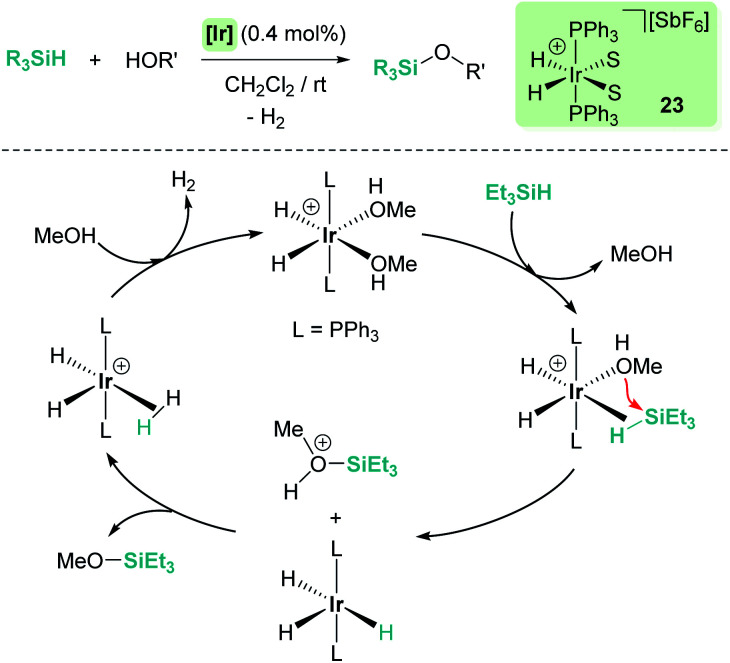
Dehydrocoupling of silanes and alcohols catalysed by 23.

Dehydrocoupling reactions of silanes and X–H bonds (X = O, N) are, together with hydrosilylation of carbonyl compounds, one of the earliest processes in which ionic outer-sphere mechanisms involving σ-SiH complexes were described. Remarkably, more than 50 years ago, Sommers *et al.* intuitively suggested that the interaction of silanes (in a way similar to what we know now for σ-SiH complexes) with the metal atoms of palladium or nickel in the metal surface of heterogeneous catalysts increases substantially the electrophilic character of the silicon atom. This fact was used to explain the inversion of the stereochemistry in dehydrocoupling reactions of chiral silanes and alcohols or amines.^48^ However, it was not until 1989 when Crabtree reported for the first time that the dehydrocoupling processes of silanes and alcohols catalysed by the well-defined iridium complex [Ir(H)_2_(S)_2_(PPh_3_)_2_][SbF_6_] (23) (S = solvent) occurred through a new mechanism based on the nucleophilic addition of alcohols to η^2^-SiH iridium intermediates (ionic outer-sphere mechanism) (Scheme 16).^49^ Several observations lead to this conclusion; first, this catalyst is not able to promote olefin hydrosilylation, something that should be possible if silyl hydride intermediates were involved in the mechanism. They concluded that olefins are not nucleophilic enough to react with the iridium σ-SiH complex. Nevertheless, ketones can undergo hydrosilylation, although at slow rates. The proposed mechanism was also supported by exhaustive kinetic studies and kinetic isotope effects. The latter, showing a *k*_OH_/*k*_OD_ value of 1.8, indicated that cleavage of the O–H bond is involved in the rate limiting step. In addition, secondary alcohols reacted faster than primary ones, in contrast to previously reported catalytic systems. The authors identified complex [Ir(H)_2_(η^2^-HSiEt_3_)_2_(PPh_3_)_2_][SbF_6_] as a likely intermediate based on low temperature NMR experiments between [Ir(H)_2_(MeOH)_2_(PPh_3_)_2_][SbF_6_] and excess of Et_3_SiH, although its instability precluded its complete characterization. Both systems were the starting point for the development of new catalysts and new procedures to forge Si–X bonds (X = O, N) based on the electrophilic activation of silanes through the formation of σ-SiH intermediates.

**Fig. 7:**
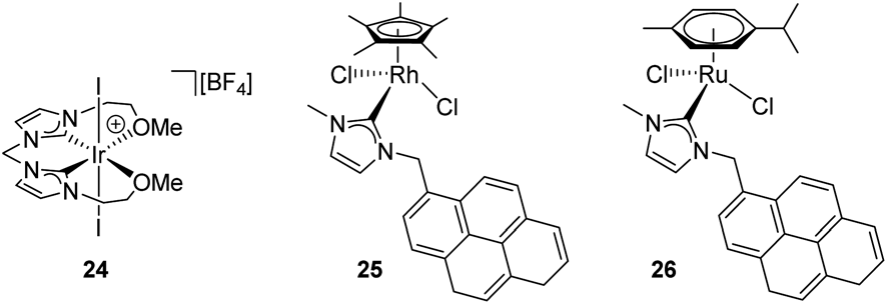
Recent examples of catalysts for dehydrocoupling processes.

Several transition metal complexes have been reported to be able to cleave the O–H bonds of alcohols or water in the presence of silanes. In most cases, the complexes are cationic, which would render a more electrophilic character to the silicon atom once coordinated. σ-SiH complexes of cationic transition metals are usually more difficult to isolate as a consequence of the large polarization that the Si–H bond undergoes, making the silicon atom more susceptible of nucleophilic attack. Therefore, the silane is more unstable towards heterolytic cleavage. For example, Brookhart *et al.* were able to characterise spectroscopically the iron complex [CpFe(η^2^-H–SiEt_3_)(CO)(PEt_3_)][BAr^F^] although it reacted with adventitious water at temperatures above −40 °C leading eventually to the the dihydrogen complex [CpFe(H_2_)(CO)(PEt_3_)][BAr^F^] and Et_3_SiOH.^50^ This behaviour was leveraged in the dehydrocoupling of ethanol or phenol with silanes leading to the corresponding silyl-ethers. Experimental evidence for the mechanism of the reaction was provided and supported by DFT calculations.^51^ Other catalytic systems for the alcoholysis or hydrolysis of Si–H bonds based on iridium complexes ([Ir(i)_2_{κC,C′,O,O′-bis(NHC^OMe^)}][BF_4_] (24),^52^ [IrCp*(Cl)_2_(NHC)] (25)^53^) and ruthenium complexes ([Ru(*p*-cym)(Cl)_2_(NHC)] (26)^54^) (Fig. 7) that are cationic or able to form transient cationic systems by dissociation of a halide have been recently reported exhibiting rather good activities (catalyst loadings between 0.1 and 1 mol%, reaction times between 2 and 90 min approx.). Some of these systems can be recycled with nearly no loss in activity. Interestingly, these catalysts operate, according to DFT calculations, through an outer-sphere mechanism that involves the participation of σ-SiH complexes in which the interaction of the Si–H bond with the metal is η^1^. However, these intermediates have neither been detected spectroscopically nor isolated.

As mentioned before, the use of cationic systems increases the reactivity of the silane towards nucleophilic attack. Nevertheless, this is not strictly necessary, as it has been shown that neutral species bearing π acceptor ligands such as CO can form σ-SiH complexes susceptible of nucleophilic attack by alcohols or water. These π acceptor ligands retrieve electron density from the metal centre, considerably reducing their ability to π back-donate into the Si–H σ* orbital. Therefore, oxidative addition processes are minimized and, at the same time, the electrophilic character at the silicon atom is increased. Examples of this kind have been reported when photochemical dissociation of CO from M(CO)_6_ (M = Cr, W) or Re_2_(CO)_10_ was performed in the presence of silanes (leading to the corresponding transient σ-SiH complexes) and alcohols.^55^

**Scheme 17:**
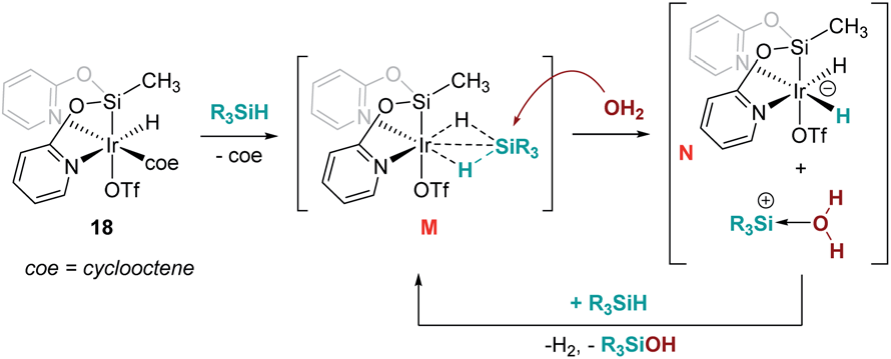
Mechanistic proposal for dehydrocoupling of silanes and alcohols induced by complex 18.

In some cases, formally neutral species not bearing π acceptor ligands have been invoked as intermediates in the cleavage of the O–H bond of water with silanes. This is the case of the iridium complex [Ir(H)(CF_3_SO_3_)(NSiN)(coe)] (18) reported by Oro, Fernández, *et al.*^56^ Counterintuitively, DFT calculations predicted that it is the coe ligand (and not the CF_3_SO_3_^−^ that would lead to a cationic intermediate) that dissociates during the catalytic process, leading to a neutral transient intermediate (not detected) [Ir(H)(HSiR_3_)(CF_3_SO_3_)(NSiN)] (M, Scheme 17) that then undergoes an S_N_2-type reaction with a molecule of water forming an anionic dihydride iridium intermediate [Ir(H_2_)(CF_3_SO_3_)(NSiN)]^−^ (N) and a water stabilised silylium ion. This step requires overcoming the highest energetic barrier of 16.4 kcal mol^−1^.

#### 3.2.2 Dehydrocoupling processes mediated by main group Lewis acids

Regarding the use of boron-based Lewis acids, ten years after the results by Crabtree were published,^49^ Piers *et al.* reported the dehydrocoupling of alcohols and silanes catalysed by B(C_6_F_5_)_3_.^57^ Stimulated by the discovery of hydrosilylation of carbonyl compounds by B(C_6_F_5_)_3_,^16^ Piers anticipated that this Lewis acid could catalyse the dehydrocoupling of silanes and alcohols and perhaps increase the tolerance to functional groups not compatible with transition metals or using bulkier silanes or tertiary alcohols under aerobic conditions. In fact, B(C_6_F_5_)_3_ proved to be an efficient catalyst, tolerant to functional groups such as double bonds, carbonyls and nitriles. However, catalyst loadings of 2 mol% were required (compared to 0.4 mol% with iridium system 23) and reaction rates were considerably slower with respect to the iridium counterpart (TOF values for iridium ranging from 340 to 130 000 h^−1^*vs.* 2 to 50 h^−1^ for B(C_6_F_5_)_3_). In both cases, secondary alcohols reacted at faster reaction rates than primary alcohols, in spite of the more basic character of the latter.

**Fig. 8:**
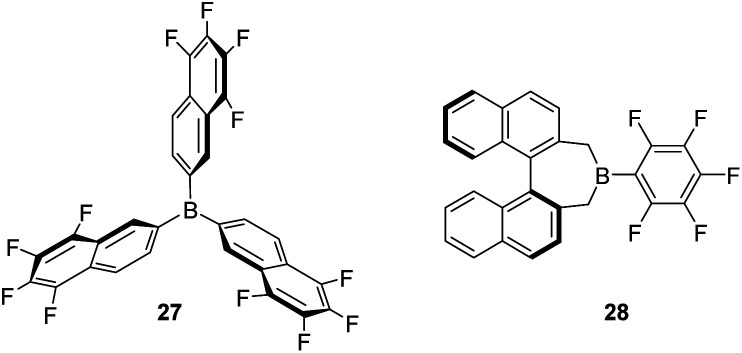
Modification of boranes for dehydrocoupling processes.

Some modifications of the structure and electronic properties were investigated by Oestreich *et al.* in order to improve its catalytic performance and to explore the effect of distal electron-withdrawing groups in aromatic rings. To this end, tris(5,6,7,8-tetrafluoronaphthalen-2-yl)borane (27, Fig. 8) was synthesised.^58^ The authors observed that the Lewis acid character of this compound is nearly identical to that of B(C_6_F_5_)_3_ and thus, it is also able to catalyse the dehydrocoupling of 1-phenylethanol and Me_2_PhSiH, suggesting that distal fluorine atoms on the structure of the borane are not detrimental to its reactivity. Although no improvement was observed with respect to B(C_6_F_5_)_3_, this study points to new possibilities in the tuning of the steric and electronic properties of boranes. In contrast, the combination of a binaphthyl backbone together with a C_6_F_5_ substituent on the boron atom (borane 28) led to a decrease in acidity to 74–85% that of B(C_6_F_5_)_3_, and resulted in a less stable catalytic system. Apparently, the cleavage of the B–C_6_F_5_ in the presence of alcohols competes with Si–H bond activation, thus higher catalyst loadings (5 mol%) are required.^59^

### 3.3 Dehydrocoupling processes of amines and silanes leading to Si–N bonds

#### 3.3.1 Dehydrocoupling processes catalysed by boron Lewis acids

**Scheme 18:**
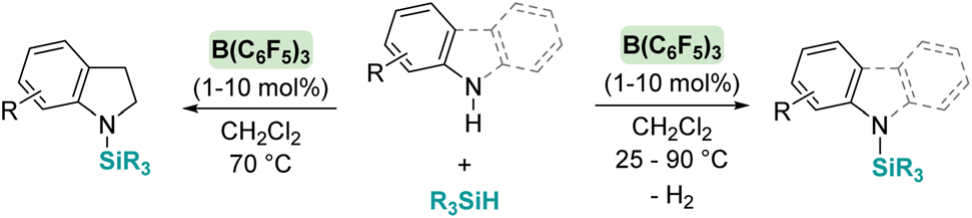
Dehydrocoupling of silanes and aromatic amines, indoles and carbazoles catalysed by B(C_6_F_5_)_3_.

The dehydrocoupling processes are not limited to the use of alcohols or water. This reactivity has also been exploited for the construction of Si–N bonds using either boron- or transition metal-based Lewis acid catalysts. Paradies *et al.* used B(C_6_F_5_)_3_ in the dehydrocoupling of silanes and aromatic amines, indoles or carbazoles under mild conditions (temperatures between 25 and 90 °C, Scheme 18).^60^ The process works well with both primary and secondary amines, but harsher reaction conditions (heating) are required for the former. This difference seems to be related to the necessity to cleave the B–N bond in the adduct formed between the amine used as a substrate and B(C_6_F_5_)_3_. In fact, less basic primary amines (such as anilines) react faster and the reaction can be carried out at room temperature. This behaviour is similar to that observed for alcohols (*i.e.* less basic alcohols react faster with silanes). Indoles can also be used, but in this case the process is accompanied by hydrogenation of the indole double bond leading to indolines (Scheme 18, left). Additionally, Zhao *et al.* calculated the energy barriers required for the N–H silylation for aliphatic amines such as morpholine or diethylamine and found that B(C_6_F_5_)_3_ is unlikely to catalyse the dehydrocoupling of these substrates since this is energetically unfavourable (requiring 33.3 and 36.4 kcal mol^−1^, respectively).^61^ These high energy barriers are associated with the formation of very stable Lewis adducts between these basic amines and B(C_6_F_5_)_3_, and might explain why no reports for the dehydrocoupling of basic amines and silanes by B(C_6_F_5_)_3_ have been published.

**Scheme 19:**
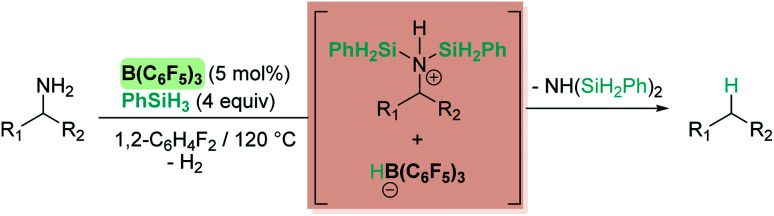
Reductive deamination catalysed by B(C_6_F_5_)_3_.

Oestreich and co-workers observed that B(C_6_F_5_)_3_ also catalyses the dehydrocoupling of benzylic amines and silanes. Very interestingly, upon heating the reaction mixture to 120 °C a reductive deamination process takes place, leading to the cleavage of the benzylic C–N bond and to a defunctionalised hydrocarbon (Scheme 19).^62^

#### 3.3.2 Dehydrocoupling processes catalysed by transition metals

**Scheme 20:**
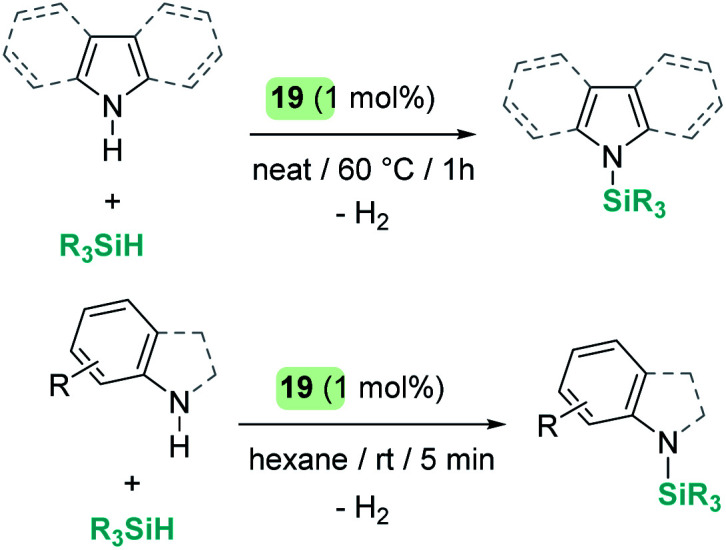
Cross-dehydrocoupling of amines and silanes catalysed by 19.

A parallelism between B(C_6_F_5_)_3_ and some transition metal-catalysed processes can be found. The group of Oestreich has been particularly involved in exploring the reactivity of B(C_6_F_5_)_3_ and late transition metals. The ruthenium system 19 has been particularly prolific as a catalyst in this area. The cooperative role of the sulfur atom upon coordination of the Si–H bond through the hydrogen atom to the ruthenium centre is a key feature of this exceptional catalytic system^63^ that has been used in dehydrocoupling of anilines, indoles, carbazoles and pyrroles (Scheme 20).^64^ Generally, the process works using 1 mol% of the ruthenium catalyst at temperatures ranging from room temperature (anilines) to 60 °C (indoles, pyrroles) in nearly quantitative yields. In line with the observations reported by Zhao on the B(C_6_F_5_)_3_ catalyst,^61^ more basic substrates, such as alkylamines, do not seem to be compatible with the catalyst due to inhibition by coordination to ruthenium.

**Scheme 21:**
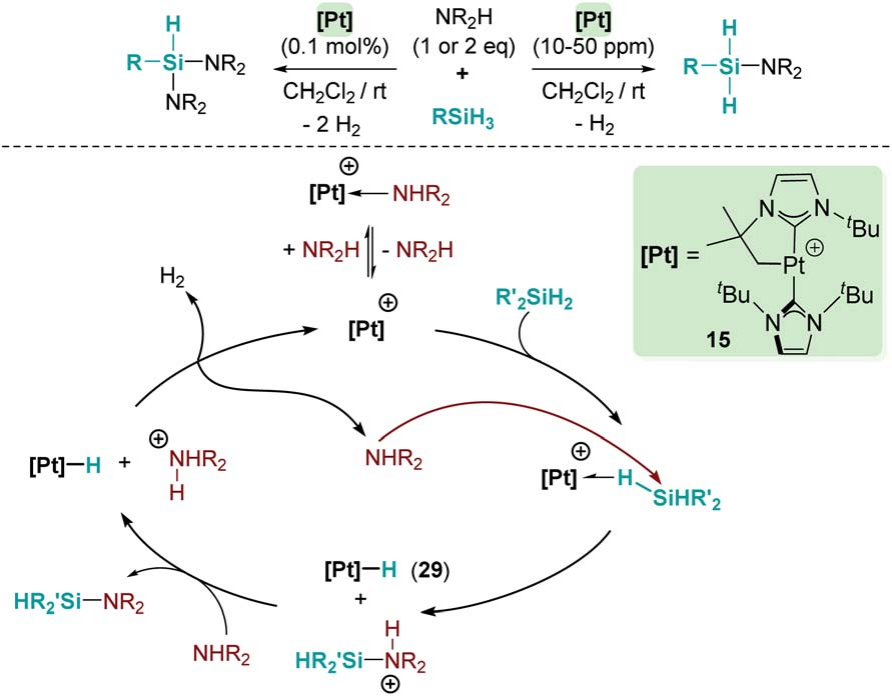
Cross-dehydrocoupling of amines and silanes catalysed by 15.

Our group has also been involved in the synthesis of silazanes *via* dehydrocoupling of amines and silanes by electrophilic platinum complexes.^65^ The cationic electron-deficient platinum complex [Pt(I^*t*^Bu′)(I^*t*^Bu)][BAr^F^], 15, proved to be an excellent catalyst for this process using alkyl-amines and silanes under mild reaction conditions (room temperature) (Scheme 21). Very low catalyst loadings at the ppm level (as low as 10 ppm) can be used when primary silanes are used. In addition, a control of the selectivity of the reaction can be achieved, leading to either monosilazanes or disilazanes by using one or two equivalents of amine. According to NMR studies and previous results, this system is able to bind silanes to form the corresponding σ-SiH complex, in which the most favoured coordination mode is η^1^.^12^ Low temperature NMR studies of the stoichiometric reaction of Ph_2_SiH_2_, HNEt_2_ and complex [Pt(I^*t*^Bu′)(I^*t*^Bu)][BAr^F^] (15) allowed the detection of the neutral platinum hydride [Pt(H)(I^*t*^Bu′)(I^*t*^Bu)] (29), monosilazane Ph_2_SiH(NEt_2_) and the ammonium cation [H_2_NEt_2_][BAr^F^]. The latter is presumed to be formed by deprotonation of a transient, not detected, silylium-stabilised cation [Ph_2_SiH(HNEt_2_)][BAr^F^] by another molecule of HNEt_2_ (Scheme 21). At higher reaction temperatures, the hydride complex 29 reacts with [H_2_NEt_2_][BAr^F^] releasing H_2_, and HNEt_2_ enters the catalytic cycle again. Therefore, at variance with the reactions with B(C_6_F_5_)_3_ and the ruthenium complex 19, basic aliphatic amines are suitable substrates for the dehydrocoupling.

**Scheme 22:**
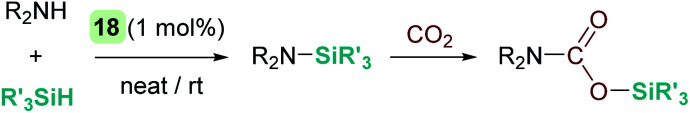
Synthesis of silyl carbamates catalysed by 18.

Oro, Fernández-Álvarez *et al.* reported as well that the iridium complex [Ir(H)(CF_3_SO_3_)(NSiN)(coe)] (18) catalyses the cross dehydrocoupling of silanes and amines leading to silazanes that in the presence of CO_2_ produce silyl-carbamates (Scheme 22).^66^ The catalytic process takes place using 1 mol% of the iridium complex at temperatures between room temperature for aliphatic amines and 45 °C for aromatic amines. It is worth noting that benzylic amines have been utilized, and no reductive deamination, as when B(C_6_F_5_)_3_ is used, has been observed. Another relevant feature of this system is that, according to DFT calculations, the reaction takes place again through a formally neutral iridium intermediate, in which the σ-SiH ligand preferably adopts an η^1^ coordination mode. The nucleophilic addition of the amine to the σ-SiH complex has been determined to be the rate determining step, requiring energetic barriers of 18.9 kcal mol^−1^ (for the least hindered amine HNMe_2_) or 21.9 kcal mol^−1^ (for the bulkier HN^i^Pr_2_).

**Scheme 23:**
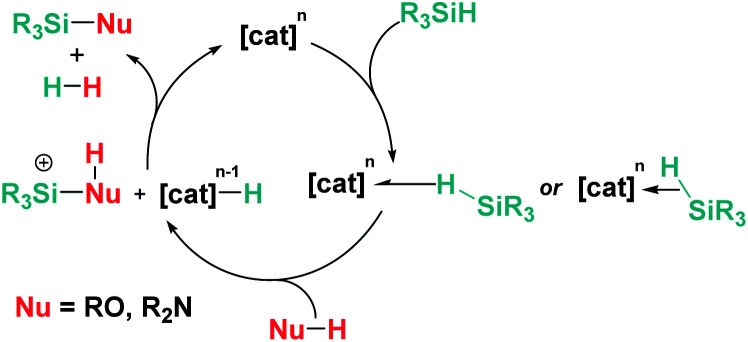
General common mechanism for dehydrocoupling processes induced by transition metals and main group Lewis acids.

Overall, the reactivity described in Sections 3.2 and 3.3 suggests that, in most cases, both main group Lewis acids and transition metal complexes follow analogous mechanistic pathways. Nevertheless, in some cases, the deprotonation of the acidic N–H proton of the transient silylium cation might take place by a second amine, and not by the metal hydride as depicted in Scheme 21 (Scheme 23).

### 3.4 C–O bond cleavage processes

**Scheme 24:**
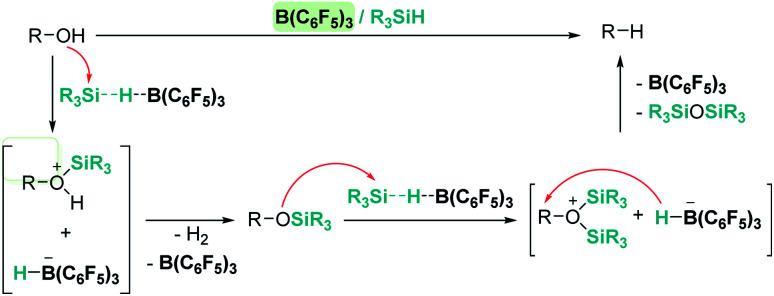
Reduction of alcohols mediated by B(C_6_F_5_)_3_.

**Scheme 25:**
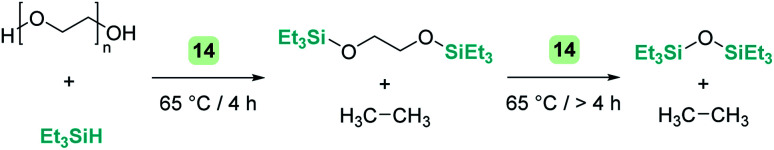
Reduction of poly(ethylene)glycol catalysed by 14.

**Scheme 26:**
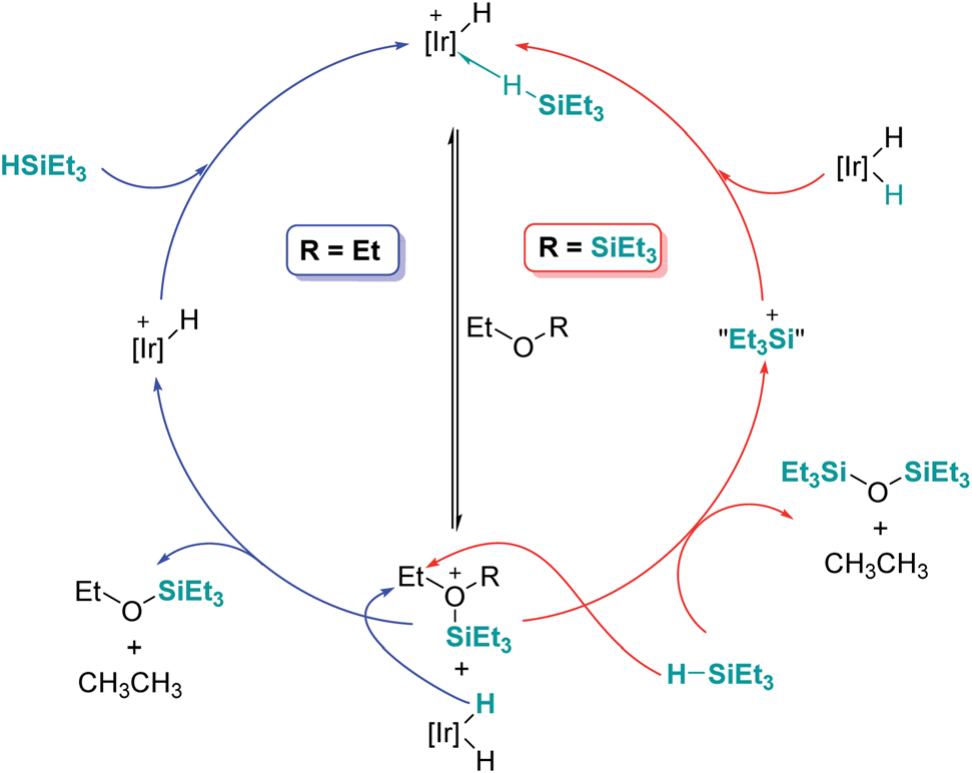
Mechanistic proposals for the reduction of ethers.

An interesting reactivity of these Lewis acid catalysts is related to their ability to cleave C–O bonds of a variety of functional groups in organic substrates. Most of the catalysts used in this particular process have been developed by using boranes or iridium catalysts. Seminal work by Gevorgyan, Yamamoto and co-workers demonstrated the efficiency of B(C_6_F_5_)_3_ to reduce alcohols and ethers to hydrocarbons in the presence of an excess of Et_3_SiH (Scheme 24).^67^ The sequence of reactions that take place for both alcohols and ethers shares in common the nucleophilic addition of the oxygen atom to the activated silane by B(C_6_F_5_)_3_. An initial dehydrocoupling reaction between the OH and the SiH leading to silyl ether is a prior step before C–O cleavage when alcohols are used. The reaction has some limitations, since aromatic alcohols do not undergo C–O bond cleavage. Secondary and tertiary alcohols cannot be reduced with Et_3_SiH, but McRae showed that their reduction can be successfully achieved using more reactive, smaller silanes such as Et_2_SiH_2_ or ^*n*^BuSiH_3_, in combination with B(C_6_F_5_)_3_.^68^ As mentioned in the previous section, it is interesting to compare this catalytic system to others that can promote the dehydrocoupling of silanes and alcohols. In most cases, the reaction stops once the silyl ether is formed, and no C–O bond breaking processes have been observed, demonstrating that the extent of activation of the Si–H bond by these systems is not sufficient to undergo a nucleophilic addition from the ether oxygen atom. Nevertheless, Brookhart reported that the iridium complex [(POCOP)Ir(H)(acetone)][B(C_6_F_5_)_4_] 14 is able to cleave poly(ethylene glycol) at long reaction times, to yield Et_3_SiOSiEt_3_ and ethane using Et_3_SiH as a reducing agent at 65 °C (Scheme 25).^69^ Cantat (see below) made similar observations using lignin models,^70^ suggesting that, likely, simple alcohols would be able to be reduced with this catalytic system, although no reports have been made in this regard. It is worth comparing Brookhart's system and B(C_6_F_5_)_3_ in the cleavage of ethers: both systems behave similarly in terms of selectivity and limitations (for example, no Ar–O bond breaking is observed), although catalytic loadings for 14 are typically 1 mol%, whereas higher catalyst loadings (5–10%) are required for B(C_6_F_5_)_3_ under similar reaction conditions (temperature, silane, *etc.*). Analysis of the reaction mechanism for the reduction of diethyl ether with Et_3_SiH by the iridium system led to different conclusions regarding the hydride source. While it seems clear that the iridium hydride ([Ir]–H) (generated after nucleophilic addition of Et_2_O to Et_3_Si⋯H⋯[Ir]) is responsible for the C–O bond cleavage (see Scheme 26) leading to EtOSiEt_3_, the cleavage of the remaining Et–O bond in EtOSiEt_3_ is facilitated by EtSiH_3_ itself, probably as a consequence of steric repulsions.^69^

**Scheme 27:**
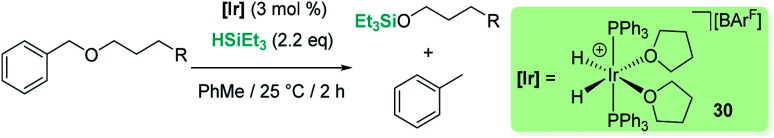
Reduction of ethers catalysed by 30.

The fact that Brookhart's iridium catalyst 14 and B(C_6_F_5_)_3_ can cleave primary, secondary and tertiary ethers can be a problem if one is dealing with organic molecules bearing these different types of C–O functional groups and selectivity is a concern for the synthesis of C–O functionalised molecules. Schley has recently shown that the iridium complex [IrH_2_(PPh_3_)_2_(THF)_2_][BAr^F^] (30) exhibits a mitigated reactivity with respect to Brookhart's iridium complex 14 in addition to different selectivity patterns (Scheme 27).^71^ Thus, no exhaustive reduction of the ethers to alkanes is observed and the catalyst is tolerant to certain ethers bearing halogenated fragments that are problematic with Brookhart's system.^34*b*,*c*^ In addition, the rupture of benzylic C–O bonds occurs preferentially over the rupture of alkyl C–O bonds (contrary to observations with 14), whereas in some cases, *e.g.* cyclopentyl methyl ether, secondary C–O bond cleavage is preferred over primary C–O bond cleavage (exactly the opposite of what is observed with B(C_6_F_5_)_3_).^72^ Thus, this system (together with related complexes with phosphines with different electronic properties) serves as an example of how systems can be modified to alter both their reactivity and selectivity. The suggested mechanism by which the reaction takes place using [IrH_2_(PPh_3_)_2_(THF)_2_][BAr^F^] (30) has been reported to follow similar pathways to those with both [(POCOP)Ir(H)(acetone)][B(C_6_F_5_)_4_] (14) and B(C_6_F_5_)_3_. However, the hydride source is not [Ir(H)_3_(PPh_3_)_2_(THF)] (formed by reaction of [IrH_2_(PPh_3_)_2_(THF)_2_][BAr^F^] with the ether and Et_3_SiH) but complex [Ir(H)_4_(SiEt_3_)(PPh_3_)_2_] (arising from oxidative addition of Et_3_SiH to [Ir(H)_3_(PPh_3_)_2_(THF)]).

**Scheme 28:**
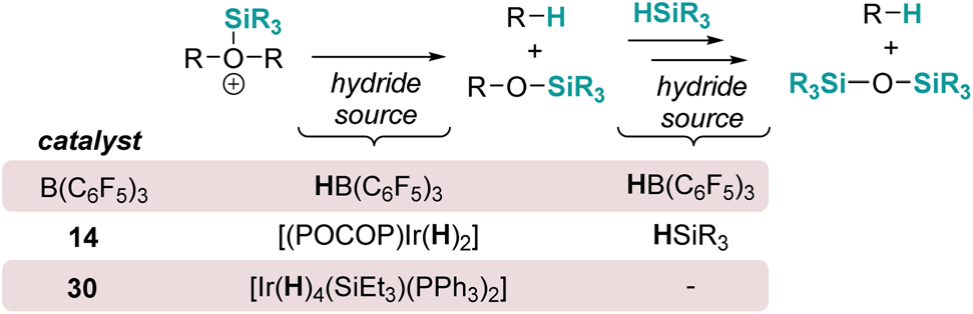
Mechanistic divergence in hydride transfer.

Concisely, all three catalysts B(C_6_F_5_)_3_, 14 and 30 share in common the first step, related to the heterolytic cleavage of the Si–H bond (as depicted in Scheme 9). However, the generated siloxonium ion reacts with different hydride sources, as summarized in Scheme 28.

**Scheme 29:**
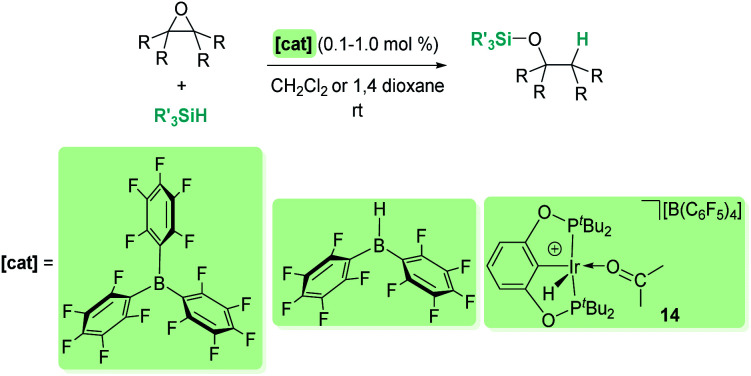
Reduction of epoxides.

Hydrosilylation of epoxides and cyclic ethers to form silyl-protected alcohols has also been explored with borane-based-Lewis acids^73^ and Brookhart's iridium complex 14.^74^ When comparing B(C_6_F_5_)_3_ and iridium, there seem to be not too many differences both in terms of reactivity (both systems operate at room temperature under similar catalyst loadings of 0.1–1 mol%) and selectivity (Scheme 29). For example, when 2,2,3,3-tetramethyloxirane is used, a secondary alcohol is the major or only component of the reaction, whose formation arises from a formal methyl migration (pinacol rearrangement). However, Chang, Park, *et al.* have observed a different selectivity when using Piers' borane HB(C_6_F_5_)_2_ as a precatalyst.^73^ This Lewis acid partially inhibits this rearrangement leading to the tertiary alcohol as the main product (20 : 1 tertiary : secondary alcohol). A careful inspection of the reaction mechanism led to a possible explanation of this different behaviour. Presumably, the true catalyst is not HB(C_6_F_5_)_2_ but an alkoxy-borane RO–B(C_6_F_5_)_2_ formed during the reaction. This new borane is able to activate the silane by the same means as, for example, B(C_6_F_5_)_3_, formally leading to a silylium ion R_3_Si^+^ (trapped by the epoxide) and the borate HB(OR)(C_6_F_5_)_2_^−^. The hydride of the latter is more reactive than the transient HB(C_6_F_5_)_3_^−^ formed when B(C_6_F_5_)_3_ acts as a catalyst. The reaction between HB(OR)(C_6_F_5_)_2_^−^ and the silyloxonium intermediate is then faster than methyl migration, thus justifying the different selectivity.

**Scheme 30:**
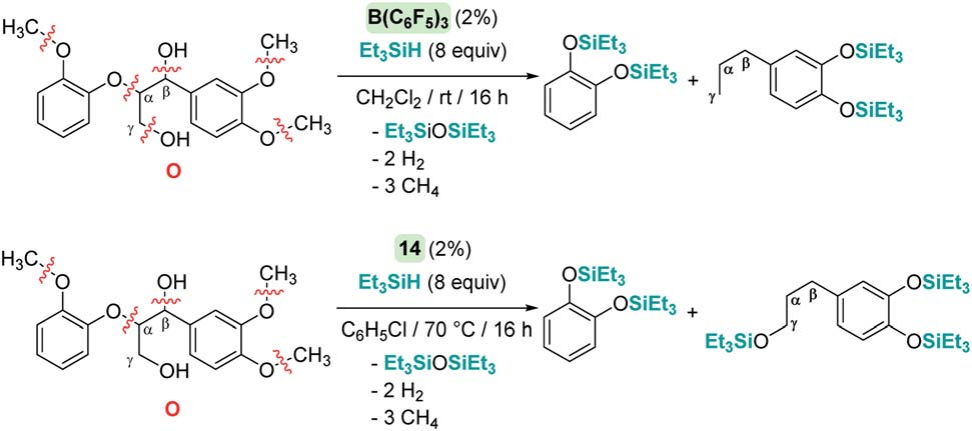
Reduction of lignin model O catalysed by B(C_6_F_5_)_3_ and 14.

The cleavage of C–O bonds by these Lewis acids has been utilised for the de-functionalisation of complex organic molecules derived from carbohydrates and lignins (and their derivatives) with two main different perspectives: the complete defunctionalisation of the molecules or the selective C–O bond cleavage. The groups of Gagné^75^ and Cantat^76^ have been particularly active in this area, with B(C_6_F_5_)_3_ being particularly dominant as a catalyst (an excellent review on the topic^77^ has been recently published). The prevalence for the utilization of B(C_6_F_5_)_3_ in this field is likely a consequence of its better performance compared to Brookhart's iridium complex 14, as well as the trend of using non-precious metal catalysts.^78^ However, Cantat observed that, in spite of the excellent activities of B(C_6_F_5_)_3_ as a catalyst for the depolymerisation of lignins to mono-aromatic compounds, the system is very sensitive to impurities present in the lignins.^76*a*^ To overcome this problem, they turned their attention to Brookhart's iridium complex 14, which exhibited a more robust behaviour and led to better selectivities in the same process.^70^ A comparison of the two catalysts in the degradation of lignin models (Scheme 30) provided some clues about the different selectivities observed under similar reaction conditions. B(C_6_F_5_)_3_ operates at room temperature, whereas iridium needs heating to 70 °C. Neither of the systems are able to cleave C(sp^2^)–O bonds but while B(C_6_F_5_)_3_ cleaves all C(sp^3^)–O bonds of model molecule O after 16 h, the iridium system is not able to cleave the C_γ_–O bonds (Scheme 30).

### 3.5 Si–C coupling reactions

#### 3.5.1 Hydrosilylation of alkenes and alkynes catalysed by main group Lewis acids

The hydrosilylation of carbon–carbon multiple bonds was one of the first processes in which a boron based reagent (B(C_6_F_5_)_3_) was first identified as a catalyst operating through an outer-sphere mechanism. In 2002, Gevorgyan reported that B(C_6_F_5_)_3_ catalyses the *trans*-selective hydrosilylation of terminal and internal olefins at room temperature using 5 mol% of the catalyst.^79^ Unlike other traditional Lewis acids, B(C_6_F_5_)_3_ is not involved in the polymerisation of styrene or indene. This observation, together with the absence of a kinetic isotope effect when deuterated silanes are used (*k*_H_/*k*_D_ = 0.96), led the authors to conclude that the most likely mechanism involves a nucleophilic attack of the silicon atom of the coordinated Si–H bond by the olefin.

**Scheme 31:**
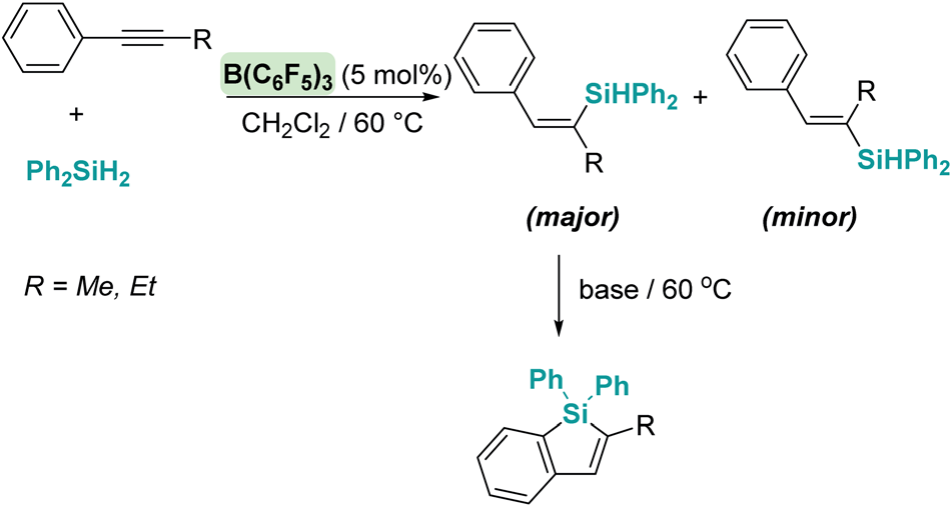
Hydrosilylation of alkynes catalysed by B(C_6_F_5_)_3_.

**Scheme 32:**
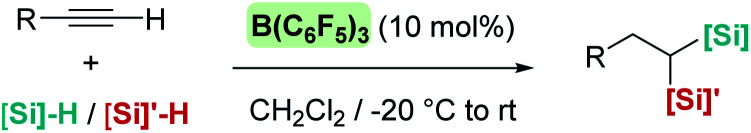
Double hydrosilylation of alkynes catalysed by B(C_6_F_5_)_3_.

**Scheme 33:**
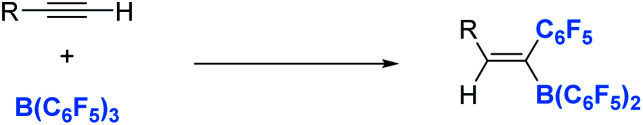
1,1-carboboration of alkynes and B(C_6_F_5_)_3_.

A few years later, Ingleson *et al.* showed that alkynes can undergo *trans*-hydrosilylation with secondary silanes to form vinyl-silanes. The pre-oriented *cis*-disposition of the remaining Si–H bond of the latter and the arene allowed, in a subsequent step, the formation of siloles (Scheme 31). However, the substrate scope was very limited.^80^ In his article, Ingleson observed that terminal alkynes could not be hydrosilylated by Ph_2_SiH_2_ in the presence of catalytic amounts of B(C_6_F_5_)_3_. Nevertheless, Li and co-workers have recently addressed this problem, and found that terminal alkynes can undergo a double hydrosilylation reaction in a highly efficient process leading to geminal bis(silanes) (Scheme 32) using a particular set of combinations of hydrosilanes.^81^ In this work, the authors noticed that hydrosilylation reactions proceed more efficiently at low temperatures (typically at −20 °C). After a cautious inspection of the process, they observed that B(C_6_F_5_)_3_ degrades through an irreversible 1,1-carboboration reaction as depicted in Scheme 33, a process that, according to DFT calculations, might take place at moderate temperatures (with a highest activation barrier of 21.6 kcal mol^−1^). However, the highest energy barriers for hydrosilylation catalysed by B(C_6_F_5_)_3_ are slightly lower (18.7 kcal mol^−1^ for the hydrosilylation of the alkyne and 20.3 kcal mol^−1^ for the vinyl-silane generated in the first step). Therefore, working at a relatively low temperature suppresses the degradation of B(C_6_F_5_)_3_ while allowing the system to overcome the energy barrier for hydrosilylation. Once this problem was identified, the authors succeeded in carrying out a sequential double hydrosilylation of a variety of aromatic and aliphatic alkynes using a combination of tertiary and primary silanes. Although the substrate scope is rather large, the presence of carbonyl groups represents a drawback, since they are also completely reduced to the corresponding alkanes (as mentioned in the previous section).

**Fig. 9:**
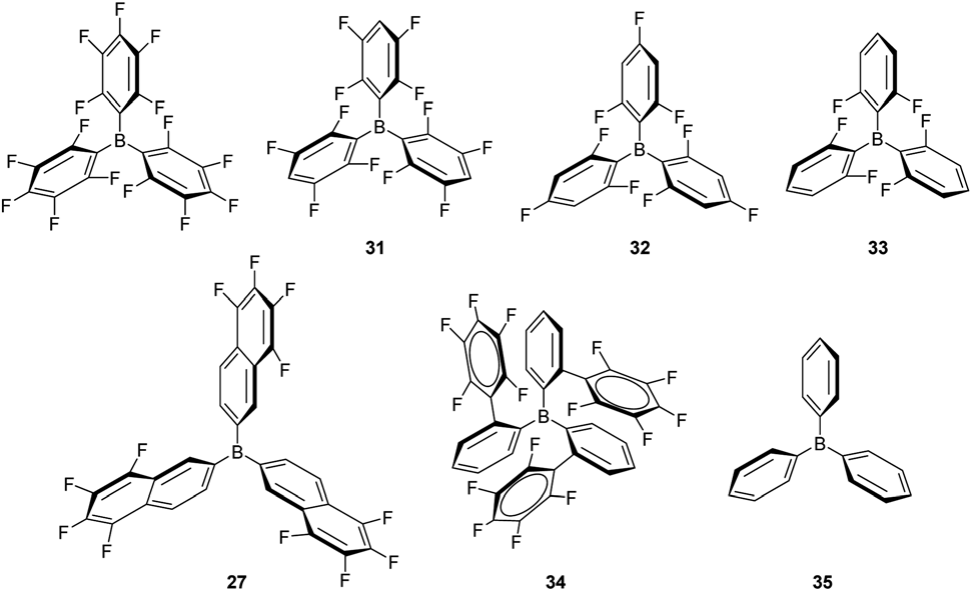
Structural modification of borane-based Lewis acids.

Although B(C_6_F_5_)_3_ is by far the most utilised boron-based catalyst, there have been some attempts to modify its structure in order to improve its chemical stability and reactivity. Oestreich *et al.* explored the reactivity of several fluorinated aryl-substituted boranes as well as triphenyl borane (Fig. 9).^82^ In their article, cyclohexa-2,5-dien-1-yl-substituted silanes were used as surrogates of silanes R_3_SiH and the Lewis acidity of boranes B(C_6_F_5_)_3_, 27 (Fig. 8) and 31–35 analysed (Fig. 9). As expected, the lower the number of fluorine atoms in the borane the lower its acidity, following the trend B(C_6_F_5_) > 31 > 32 > 33 > 35. On the other hand, boranes 27 and 34 are more or equally acidic, respectively, compared to B(C_6_F_5_). Still, B(C_6_F_5_)_3_ and 31 were the only systems able to catalyse the hydrosilylation of 1,2-diphenylacetylene and 1,1-diphenylethylene. Borane 34 showed no catalytic activity, likely as a consequence of steric effects that preclude coordination of the silane in the expected η^1^-fashion. However, borane 27 should be less sterically congested and therefore other factors must be taken into account. According to the authors, the fluorine atoms at the *ortho* position of the borane in B(C_6_F_5_) (and 31) can interact with the silicon atom, inducing an increase in its electrophilicity in the B(C_6_F_5_)_3_⋯H–SiR_3_ complex, an interaction that cannot take place with 27.

**Scheme 34:**
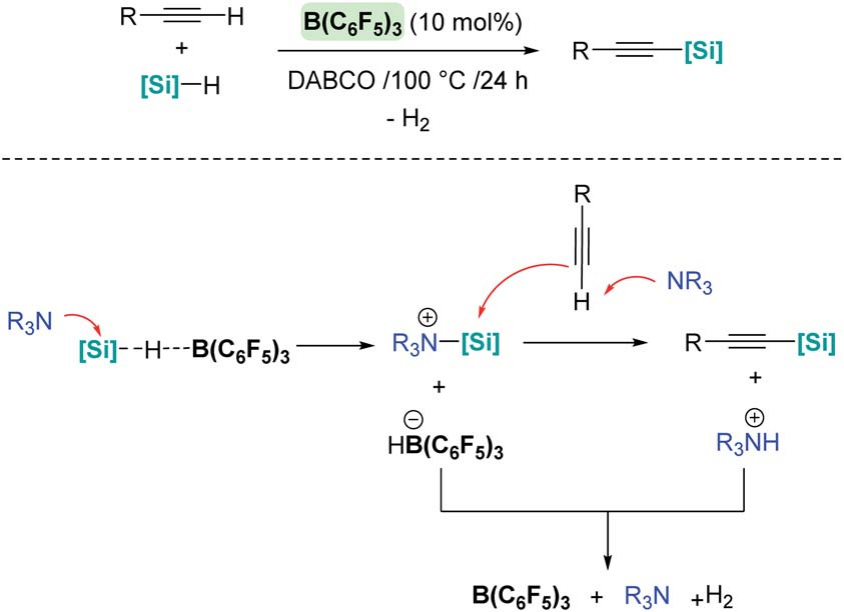
Silylative dehydrocoupling of alkynes.

It is noteworthy that upon changing the reaction conditions B(C_6_F_5_)_3_ can catalyse the silylative dehydrocoupling of terminal alkynes and silanes (C_(sp)_–H silylation), as reported by Hou, Luo, *et al.* (Scheme 34).^83^ In this case, the role of the base is of paramount importance, since no reaction is observed in its absence. According to mechanistic studies and DFT calculations, the most likely mechanism involves an initial nucleophilic attack of the silicon atom in the σ-SiH complex by the base (Scheme 34, bottom). The newly formed silylium-like cation (stabilised by the base) undergoes a nucleophilic addition of the alkyne assisted by a second molecule of base, leading to the silylated alkyne and the ion pair [(C_6_F_5_)_3_BH]^−^[HNR_3_]^+^. The latter undergoes H_2_ release, regenerating the catalyst and the base, which is the rate limiting step (Δ*G* = 32.5 kcal mol^−1^).

**Scheme 35:**
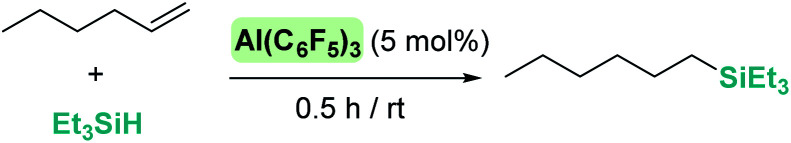
Hydrosilylation of 1-hexene catalysed by Al(C_6_F_5_)_3_.

Some attempts have been made at exploring the Lewis acidity of aluminium-based catalysts in the hydrosilylation of C–C multiple bonds. Here, alternative mechanisms other than the simple outer-sphere electrophilic activation of silanes can be anticipated. Among them, insertion of C–C multiple bonds into Al–H hydrides followed by metathesis of Al–C/Si–H bonds,^84^ or activation of the multiple bonds by the aluminium acidic center are possible reaction pathways.^85^ For the sake of simplicity, we will describe only those systems in which the authors have suggested the electrophilic outer-sphere mechanism to better explain their observations. An earlier report by Yamamoto *et al.* in 1990 proposed that activation of ClMe_2_SiH by AlCl_3_ is the first step in the hydrosilylation of 1-methylcyclohexene, followed by nucleophilic attack of the double bond, to explain the *cis* stereoselectivity of the final products.^86^ More recently, Chen reported that Al(C_6_F_5_)_3_ can mediate the hydrosilylation of the unactivated alkene 1-hexene with an efficiency considerably superior to that of B(C_6_F_5_)_3_: at the same catalyst loading of 5 mol%, Al(C_6_F_5_)_3_ converts 1-hexene into the silyl-alkane in 98% yield after 0.5 h *vs.* 92% in 12 h by B(C_6_F_5_)_3_ at the same temperature (Scheme 35).^17^

**Scheme 36:**
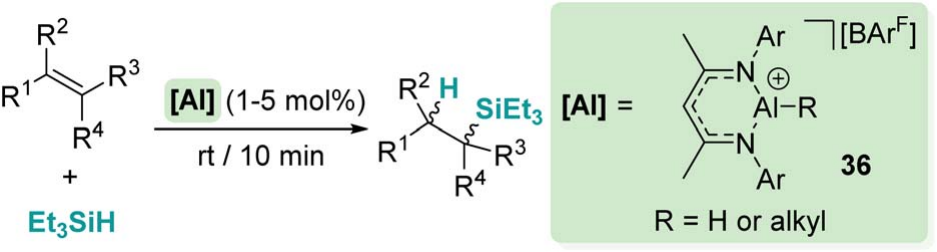
Hydrosilylation of alkenes catalysed by 36.

**Scheme 37:**
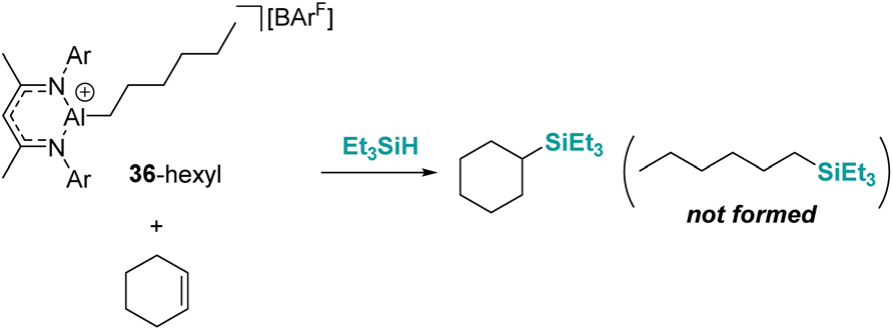
Stoichiometric hydrosilylation of cyclohexene by 36-hexyl.

On the other hand, Nikonov *et al.* have shown that hydrosilylation of olefins induced by the cationic NacNac derivative 36 (Scheme 36) is possible.^84^ The reaction is very efficient, working with as low as 1 mol% of the catalyst in short reaction times for mono- and di-substituted alkenes, although higher catalyst loadings are required for tri- and tetra-substituted olefins (5 mol%). The authors point to an outer-sphere nucleophilic addition of the alkene (or alkyne) to the R_3_Si⋯H⋯[Al]^+^ complex to explain some observations. First, there is ligand redistribution in the final product during hydrosilylation of 3,3-dimethyl-1-butene (which forms (2,3-dimethylbutyl)triethylsilane). Secondly, no hexyl-silylated products are formed in the stoichiometric reaction of complex 36-hexyl, cyclohexene and triethylsilane (Scheme 37).

#### 3.5.2 Hydrosilylation of alkenes and alkynes catalysed by transition metals

**Scheme 38:**
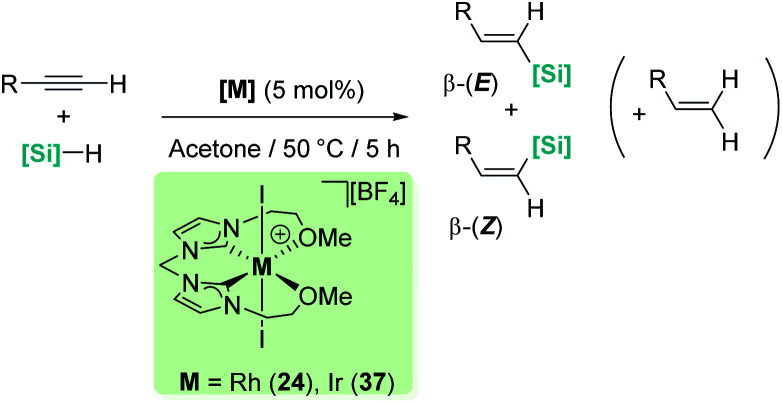
Hydrosilylation of alkynes catalysed by 24 and 37.

**Scheme 39:**
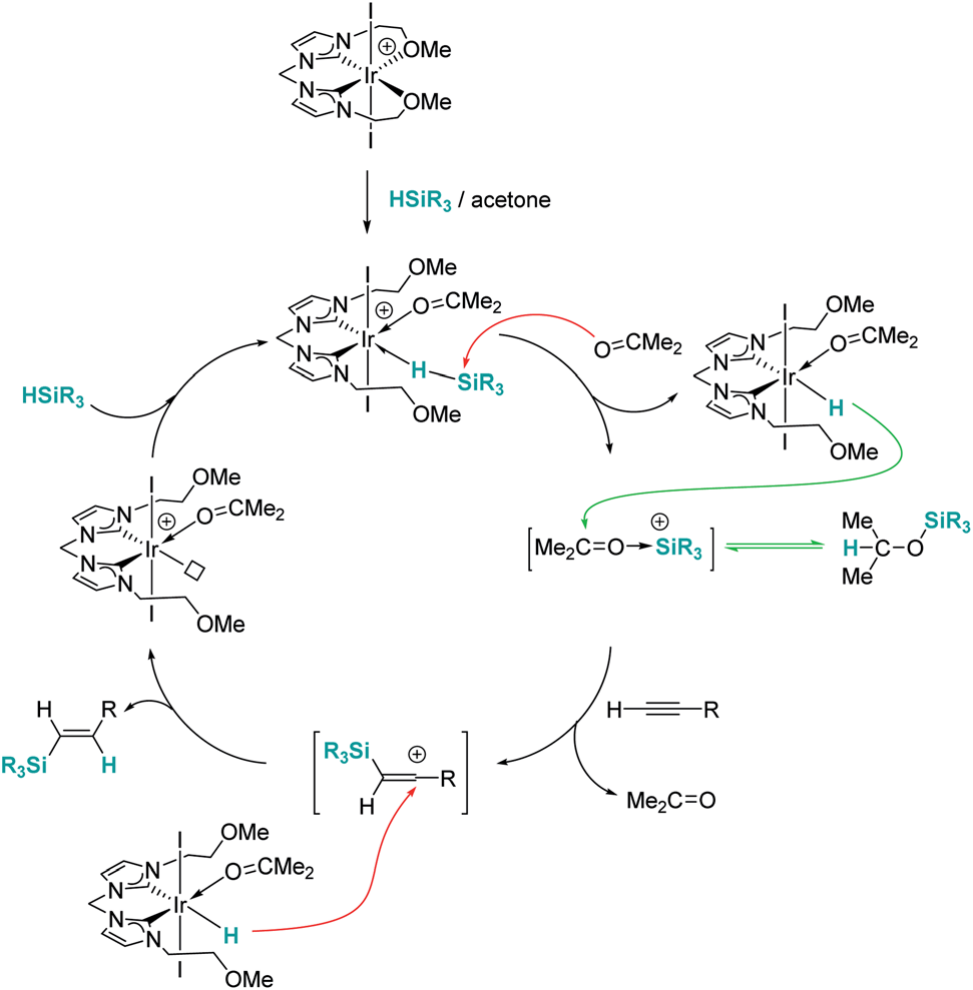
Mechanism for the hydrosilylation of alkynes catalysed by 37.

Late transition metals are known to be involved in outer-sphere nucleophilic addition into σ-SiH complexes. Iglesias, Fernández-Álvarez, Oro, *et al.* reported that cationic rhodium and iridium complexes catalyse the hydrosilylation of terminal alkynes (Scheme 38).^87^ The reaction proceeds with complete conversions leading to the β-vinyl silanes (the *Z* isomer being the major component) along with some hydrogenated alkynes (typically less than 10%). Very interestingly, the reaction does take place only in acetone, whereas other solvents such as toluene, CH_2_Cl_2_, CHCl_3_, MeOH or CH_3_CN do not work at all. Several possible mechanistic scenarios were considered for the iridium system: the classical Chalk–Harrod mechanism (or the modified version), formation of vinylidenes and an outer-sphere nucleophilic addition assisted by acetone. DFT calculations favour the last one and involve the formation of a σ-SiH iridium complex with an η^1^ coordination mode (formed after dissociation of one of the pendant OMe ligands) (Scheme 39). Thereafter, a molecule of acetone comes into play and induces the heterolytic cleavage of the Si–H bond, leading to a neutral iridium hydride and a reactive silylium-like cation stabilised by acetone. At this point, two possible reaction pathways can take place: on one hand, the transfer of the hydride from iridium to the oxo-carbenium ion is a low energy process, formally leading to the hydrosilylation of acetone. However, this process is reversible, allowing the system to evolve through an alternative reaction pathway in which the regenerated “silylium” ion undergoes nucleophilic addition of the alkyne, leading to a β-silylcarbocation that reacts with the iridium hydride, generating the β-vinylsilane. Thus, acetone is simply acting as a “silylium” transfer agent in a way not too different to the role that the amine (DABCO) has in the aforementioned dehydrocoupling of alkynes and silanes by B(C_6_F_5_)_3_ (Scheme 34).^83^

**Fig. 10:**
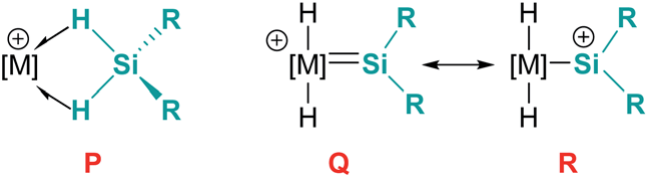
Bis(σ-SiH) coordination *vs.* double SiH bond activation.

**Scheme 40:**
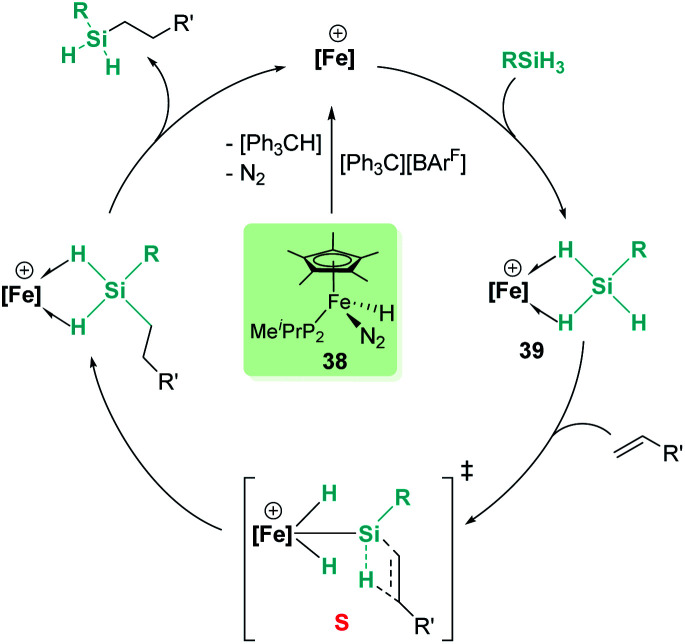
Mechanism for the hydrosilylation of alkenes catalysed by 38.

In the rhodium and iridium systems described above (24 and 37), the metal centre has two formally vacant sites that are occupied during the catalytic cycle by one molecule of acetone and one molecule of silane. The acetone molecule is a mere spectator, thus the metal atom behaves in a similar way to boron- or aluminium-based catalysts. However, some transition metals can use these two formal vacant sites to activate a silane. Some relevant examples have been reported by Tilley *et al.*, in which the silane binds the metal centre to form a bis(σ-SiH) complex (P, Fig. 10).^88^ In some cases, double Si–H activation might lead to a bis-hydride silylene complex (Q) with a strong electrophilic character as a consequence of the resonant structure R shown in Fig. 10. In any of these structural possibilities, an unsaturated C–C bond can undergo hydrosilylation, although through a distinct mechanistic pathway. Focusing on bis(σ-SiH) coordination, Tilley has recently reported that the [Cp*Fe(H)(N_2_)(P^i^Pr_2_Me)] iron complex (38) can form cationic complexes [Cp*Fe(H_2_SiHR)(P^i^Pr_2_Me)][BAr^F^] (39) in the presence of primary silanes and the hydride abstractor [Ph_3_C][BAr^F^] (Scheme 40).^89^

Once formed, these cationic systems are involved in hydrosilylation reactions of a variety of terminal and internal alkenes and some internal alkynes. The catalytic procedure involves the use of only 0.1 mol% of the catalyst at room temperature. The mechanism for this process comprises an unusual insertion of the C–C multiple bond into the Si–H bond without direct participation of the iron centre. The transition state (S, Scheme 40) for this insertion indicates that the bridging Si–H bonds are elongated (with concomitant shortening of the Fe–H bonds), which can translate into a double Si–H activation in this transition state. The rate limiting step is the exchange of the newly formed secondary silane by another equivalent of primary silane.^90^ In this regard, it is worth comparing the iron system with the ruthenium analogue [Cp*Ru(^i^Pr_3_P)(H)_2_(SiHPh)][B(C_6_F_5_)_4_]. In the latter case, the silane complex is best described as a bis-hydride silylene (Fig. 10, Q) instead of the bis(σ-SiH) calculated for the iron compound (Fig. 10, P). This difference has some important implications since dissociation of the silane requires higher energy barriers for ruthenium than for iron once the Si–C bond has been formed. In other words, the inability of iron to double activate the Si–H bonds results in a system that, while keeping an enhanced electrophilicity at the silicon centre, facilitates the dissociation of the silane in the final product necessary for turnover.

**Scheme 41:**
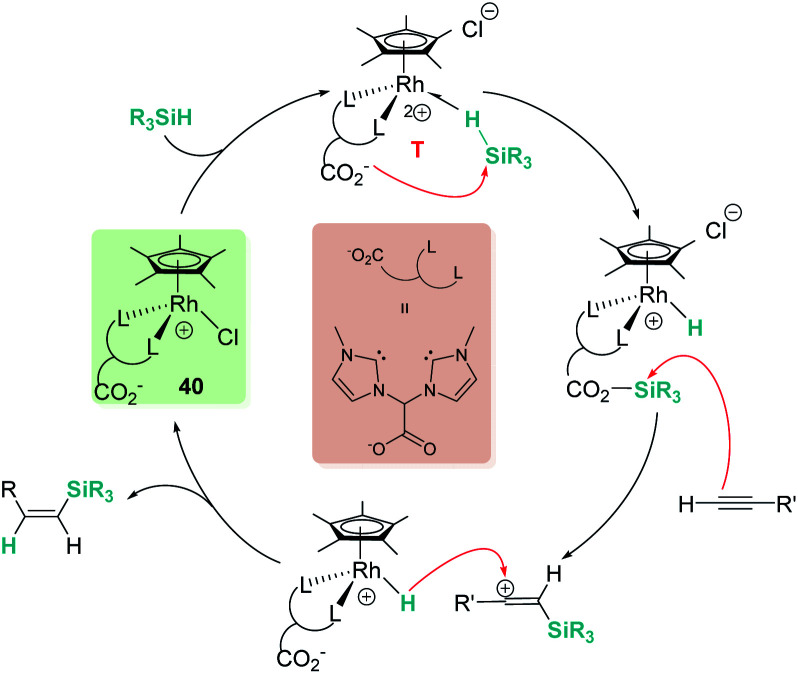
Mechanism for the hydrosilylation of alkynes by 40.

In a recent contribution, Pérez-Torrente, Jiménez, *et al.* have reported zwitterionic rhodium complex [Cp*RhCl-{(MeIm)_2_CHCOO}], 40, which catalyses the regio- and stereoselective hydrosilylation of terminal alkynes, yielding β-(*Z*)-vinylsilanes (Scheme 41).^91^ They provided evidence (by means of DFT calculations, isotopic labelling and mass spectrometry) for a catalytic system working through an outer-sphere mechanism. The carboxylate group plays a key role by temporarily trapping an “R_3_Si^+^” fragment from a σ-SiH iridium complex (intermediate T, η^1^-coordination mode) and transferring it to the terminal alkyne. The latter step generates a β-silylcarbocation intermediate that upon reacting with a Rh–H unit yields the vinylsilane. Thus, the carboxylate moiety is assisting the Si–H bond cleavage, acting as an “R_3_Si^+^” shuttle.^92^ Importantly, if the catalyst is modified by substituting the CO_2_^−^ fragment in the bis-NHC ligand by either an ester or a CH_2_ group, the catalytic activity is significantly decreased (ester) or even inhibited.

**Scheme 42:**
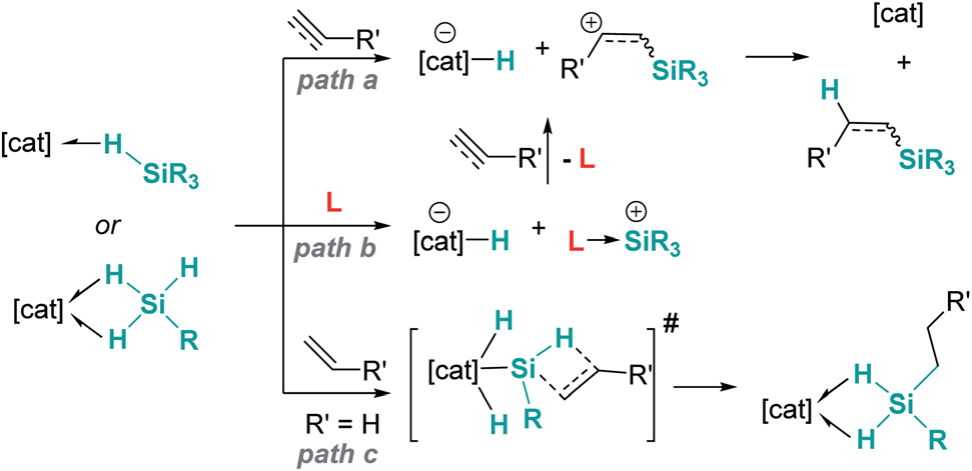
Possible reaction pathways for hydrosilylation of alkenes and alkynes.

In summary, main group and transition metal Lewis acids share some steps in the mechanism of hydrosilylation of alkenes and alkynes. As described above, both systems can catalyse these processes following the reaction pathways *a* or *b* in Scheme 42. However, for some specific transition metal complexes, such as that reported by Tilley, an alternative route (path *c*) can occur as a consequence of the ability of the metal to temporarily cleave Si–H bonds, increasing in this way the acidity of the silicon atom. This activation mode is not feasible with main group catalysts.

#### 3.5.3 Hydrosilylation of electron-rich multiple bonds and aromatic compounds

**Scheme 43:**
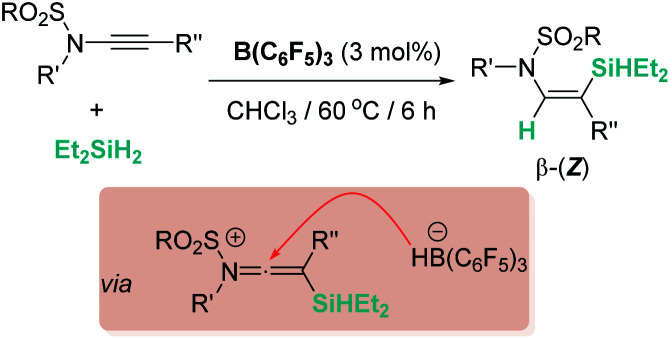
Hydrosilylation of ynamides catalysed by B(C_6_F_5_)_3_.

The hydrosilylation of electron-rich triple (such as ynamides) and double bonds (in the form of transient enamines) has also been addressed. For example, Chang *et al.* have reported that B(C_6_F_5_)_3_ catalyses the regio- and stereoselective hydrosilylation of internal ynamides under mild conditions (Scheme 43).^93^ The high nucleophilicity of the β carbon atom directs the regioselectivity to form β-silylenamide products with a *Z* stereoselectivity arising from an *anti* addition of the hydride to a keteniminium intermediate (Scheme 43).

Similar regio- and stereoselectivity (with *Z*/*E* ratios ranging from 5/1 to 20/1) have been observed using the rhodium catalyst [Rh(CO)_2_Cl]_2_.^94^ Although a mechanistic study has not been carried out, the authors proposed an outer-sphere pathway instead of a Crabtree–Ojima mechanism^95^ to better explain the results obtained with Ph_3_SiH in comparison with Et_3_SiH for some of the substrates utilised. At variance with B(C_6_F_5_)_3_, this rhodium catalyst is active at room temperature. Interestingly, it was observed that the ruthenium catalyst [CpRu(NCMe)_3_][PF_6_] is also active for this transformation, but is poor in terms of both the regio- (*β*/*α* ratio: 2.4/1) and stereo-selectivity (*Z*/*E* ratio: 6.2/1).

**Scheme 44:**
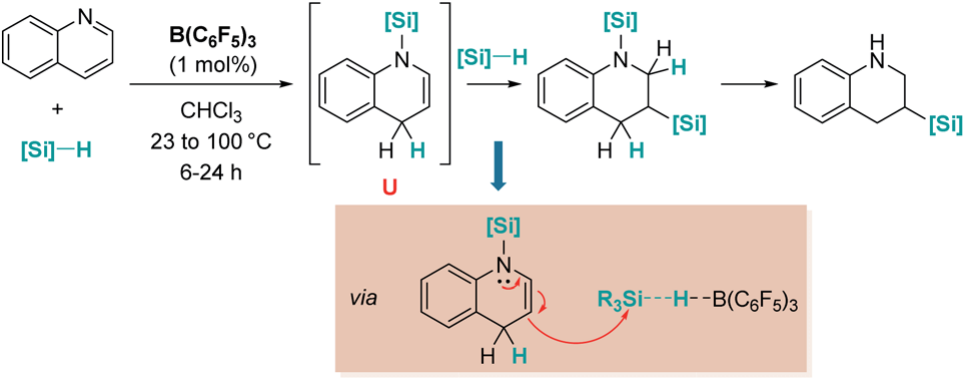
Hydrosilylation of quinolines catalysed by B(C_6_F_5_)_3_.

Electron-rich alkenes derived from enamines have not been directly used, but they have been formed as intermediates in reactions with a variety of organic substrates.^96^ One earlier report by Chang, Park, and co-workers was found in reactions of quinolines with hydrosilanes in the presence of catalytic amounts of B(C_6_F_5_)_3_ (Scheme 44).^97^ They observed that the reaction leads to the silylative reduction of quinolines to tetrahydroquinolines by a formally double hydrosilylation process. The first step involves the 1,4-addition of the silane to quinoline to afford a hydroquinoline that contains an enamine functional group (Scheme 44, U). The C3 atom of this molecule is nucleophilic and can react with a second silane equivalent, affording the bis-silylated tetrahydroquinoline through an S_N_2-type mechanism involving [R_3_Si–H⋯B(C_6_F_5_)_3_] adducts. DFT and isotopic labelling studies support this mechanism, and indicate that the first step is rate limiting, whereas the second is energetically accessible, requiring only 8 kcal mol^−1^. Interestingly, silylative reduction of pyridine or its derivatives has not been reported yet using B(C_6_F_5_)_3_.

**Scheme 45:**
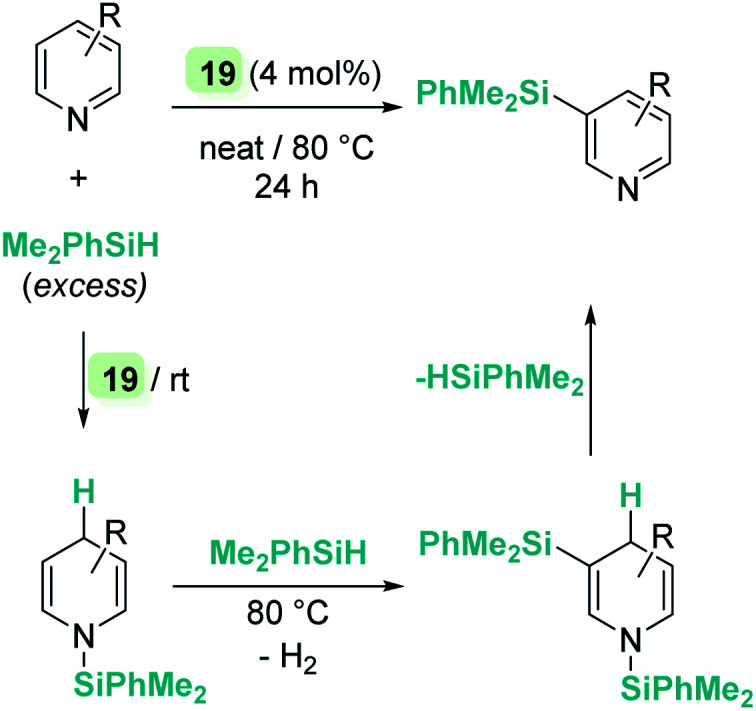
Hydrosilylation of N-heterocycles by 19.

On the other hand, quinolines and pyridines have been used as starting materials in metal-catalysed reactions with ruthenium complexes at room temperature.^98,99^ However, at variance with B(C_6_F_5_)_3_, the catalytic reaction stops after the first step (1,4-addition) and no subsequent reactivity was observed leading to formation of C–Si bonds. Nevertheless, pyridines do react in the presence of Oestreich's ruthenium catalyst 19 leading to C3-silylated pyridines under heating (Scheme 45).^100^ A close inspection of the reaction mechanism revealed that generation of the C3-silylated pyridines takes place in a three-step sequence involving a first reduction of pyridine, at room temperature, to the *N*-silylated 1,4-dihydropyridine.^98,100^ The second step requires heating at 80 °C and involves the formal transfer of a silylium cation to the nucleophilic C3 carbon atom of the 1,4-dihydropyridine, followed by a deprotonation process mediated by the ruthenium hydride intermediate. The 1,3-disilylated 1,4-dihydropyridine thus formed undergoes rearomatization, yielding the C3-silylated pyridine. The process has some limitations, working particularly well for 2-substituted pyridines, but not for pyridine itself (no reaction) or quinoline (intractable mixtures).

**Scheme 46:**
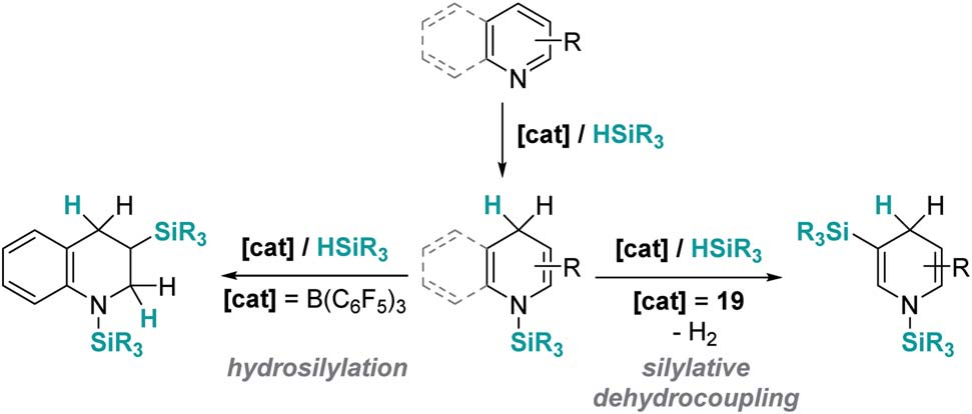
Divergent reaction pathways in the hydrosilylation of N-heterocycles.

The results described above, particularly those in Schemes 44 and 45, reveal some differences that are summarized in Scheme 46. The first step in the hydrosilylation of nitrogen-heterocycles appears to follow the same trend, leading to 1,4-hydrosilylated products. However, the addition of a second hydrosilane equivalent leads either to a hydrosilylation process (B(C_6_F_5_)_3_) or to a silylative dehydrogenation event (complex 19). Caution has to be taken since quinolines and pyridines are being compared, and it has been observed that the former are only 1,4-hydrosilylated by catalyst 19.^98*c*^

**Scheme 47:**
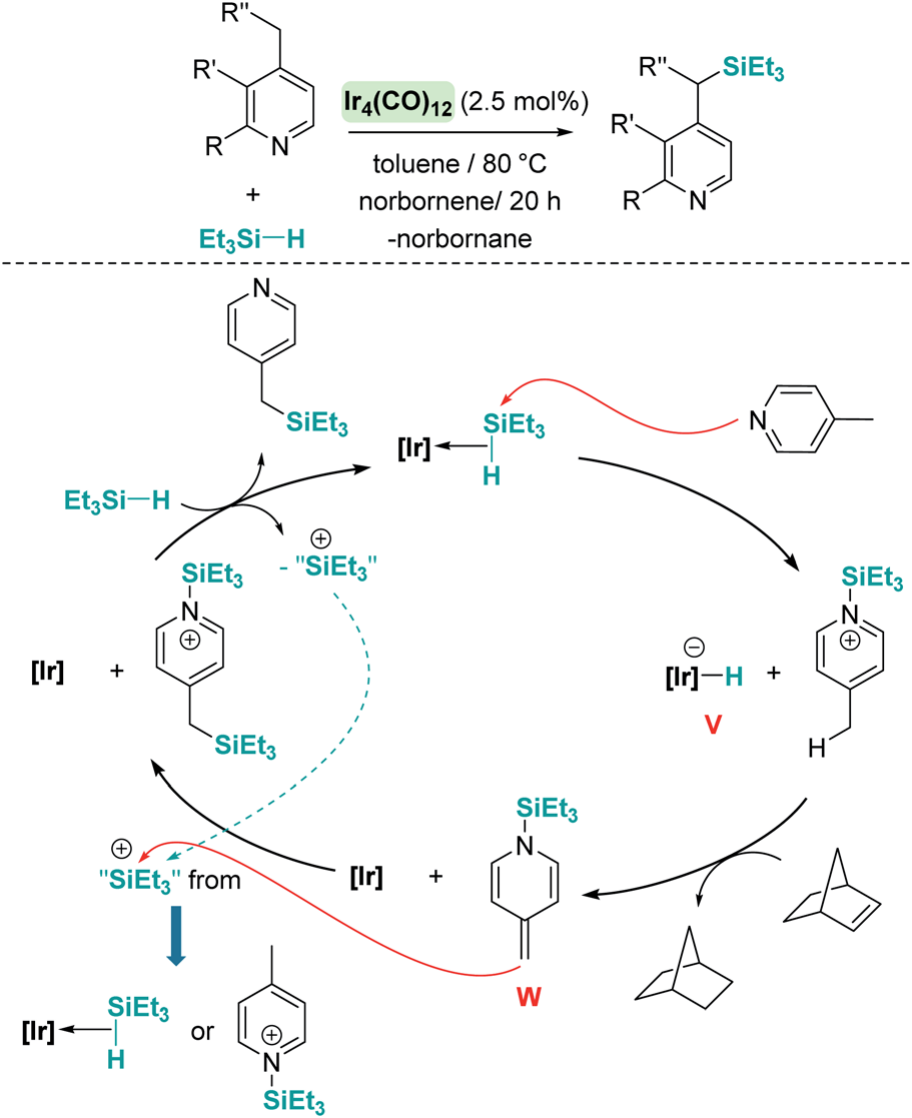
Dehydrogenative silylation of 4-alkyl pyridines catalysed by Ir_4_(CO)_12_.

A remarkable result was reported by Fukumoto when using 4-substituted alkylpyridines in the presence of neutral iridium carbonyl complexes such as Ir_4_(CO)_12_.^101^ They observed that this iridium complex catalyses selectively the C(sp_3_)–H dehydrogenative silylation of the alkyl group in the 4-position (Scheme 47). The reaction requires norbornene as a hydrogen scavenger and heating to 80 °C. The authors noticed some relevant features of the catalytic process that point to an electrophilic activation of the silane by the iridium complex assisted by the nitrogen atom. First, the reaction is sensitive to the bulkiness of the substituents at the C2-position. The bulkier the R group, the harsher the reaction conditions (R = hexyl requiring 120 °C whereas if R = ^i^Pr a temperature of 160 °C is necessary). On the other hand, 2,4,6-trimethyl pyridine does not react at all, suggesting that steric crowding around the nitrogen atom inhibits the reaction in spite of being in the opposite site to the reactivity site (benzylic position at C4). These facts, together with some isotopic labelling studies using deuterated silanes, led the authors to suggest the mechanism depicted in Scheme 47. The first step involves the transfer of a silylium fragment from an iridium σ-SiH complex (either η^1^ or η^2^) to the pyridine molecule, leading to an *N*-silylated pyridinium cation and an anionic iridium hydride (V). The presence of CO ligands is very important to prevent the oxidative addition of the σ-SiH complex and to stabilise transient hydride V. In a subsequent step, deprotonation of the alkyl group in the C4 position by the Ir–H leads to an iridium bis-hydride (that is dehydrogenated by norbornene, regenerating the iridium active species) and an *N*-silylated conjugated enamine (W). The enamine is carbon-silylated by either a newly formed iridium σ-SiH complex or by a silylium-pyridine stabilised cation. Final elimination of a “SiEt_3_^+^” fragment from the nitrogen atom furnishes the final product.

**Scheme 48:**
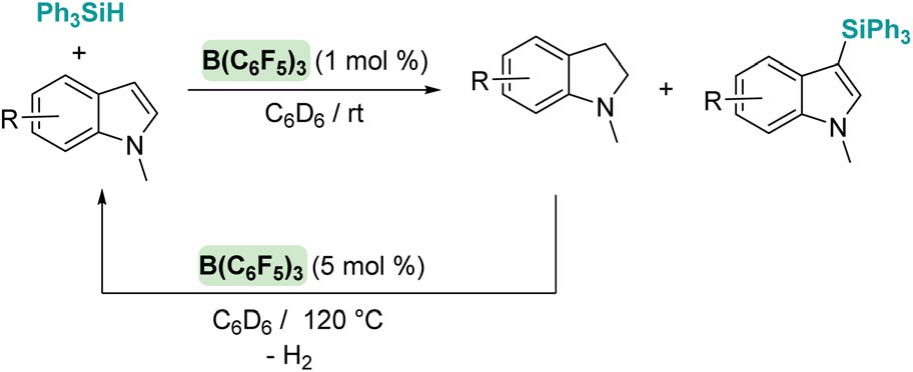
CH silylation of indoles catalysed by B(C_6_F_5_)_3_.

Silylation of indoles has been the subject of studies using p-block or ruthenium-based catalysts. A stimulating result was described by Zhang *et al.* using boron-based catalysts in the C3-silylation of indoles (Scheme 48).^102^ They reported that the dehydrocoupling of silanes and indoles can be catalysed by B(C_6_F_5_)_3_ at room temperature (other boron-based acids such as BF_3_, BCl_3_, BBr_3_, BPh_3_ or BMes_2_F do not react), yet the silylated product is formed together with equimolar amounts of the hydrogenated product (indolines). Nevertheless, indolines can be oxidised back into indoles with the same catalyst (B(C_6_F_5_)_3_) under heating^103^ and thus the reaction ratio can be directed almost exclusively to the silylated product.

**Scheme 49:**
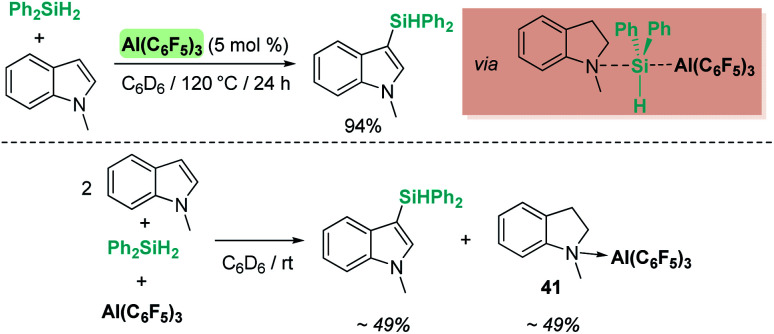
CH silylation of indoles catalysed by Al(C_6_F_5_)_3_.

The same group reported that Al(C_6_F_5_)_3_ can also catalyse the same reaction (Scheme 49), although some differences have been observed.^104^ For example, if the reaction is carried out at room temperature, the silylated indole and the indoline·Al(C_6_F_5_)_3_ adduct (41) are formed in minor quantities, with 95% of the indole recovered intact. If the reaction is performed using a 2 : 1 : 1 ratio of indole : silane : Al(C_6_F_5_)_3_, complete indole conversion is observed with formation of the silylated indole and the aluminium·indoline adduct in 49% yield each (Scheme 49, bottom). Another important difference is that Al(C_6_F_5_)_3_ does not catalyse the dehydrogenation of indoline to indole (even under heating at 120 °C). However, if the reaction is carried out at 120 °C full conversion to the silylated indole is observed. After a careful analysis of the reaction mechanism, the authors concluded that the true active catalyst is the indoline·Al(C_6_F_5_)_3_ adduct 41, that is behaving as a “thermally induced frustrated Lewis pair” activating the silane as shown in Scheme 49. In fact, higher yields of the silylated indoles can be obtained when directly using the indoline·Al(C_6_F_5_)_3_ adduct as a catalyst. It was also determined that amines that bind strongly to Al(C_6_F_5_)_3_ do not catalyse the reaction, supporting the fact that indoline has to dissociate from aluminium to activate the silane.

**Scheme 50:**
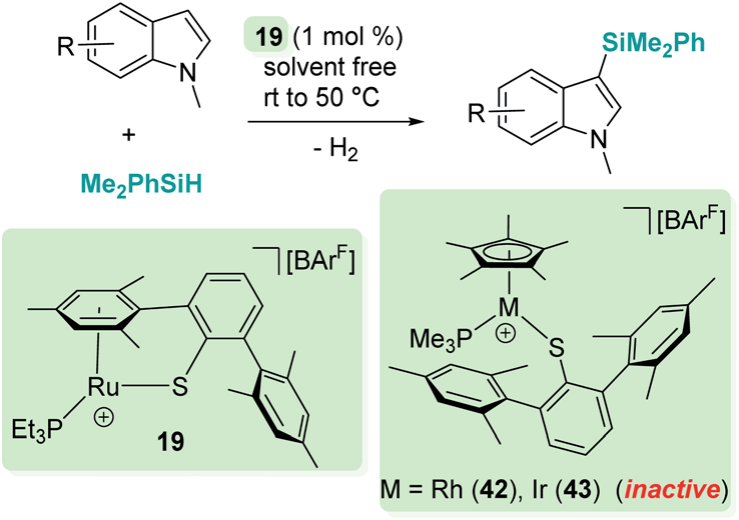
CH silylation of indoles catalysed by 19.

Regarding the use of transition metals, Oestreich, Tatsumi, Ohki, *et al.* found that the ruthenium complex 19 is very active in the C3 dehydrogenative coupling of indoles (Scheme 50).^105^ The related rhodium (42) and iridium (43) complexes were not active at all. Catalyst 19 can be used with loadings up to 0.1 mol% and recycled up to 10 times without losing its activity significantly. At variance with B(C_6_F_5_)_3_ and Al(C_6_F_5_)_3_, the reaction proceeds under mild reaction conditions (from room temperature to 50 °C in 2–6 h) and no indolines derived from hydrogenation of the indoles were detected.

**Scheme 51:**
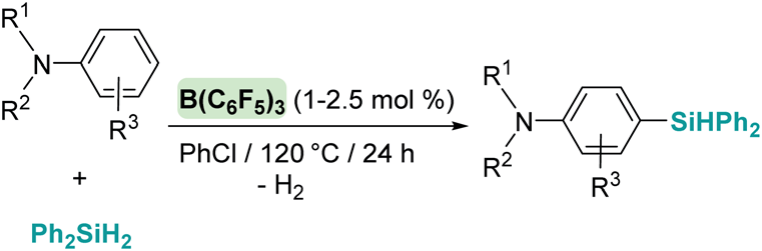
CH silylation of anilines catalysed by B(C_6_F_5_)_3_.

**Scheme 52:**
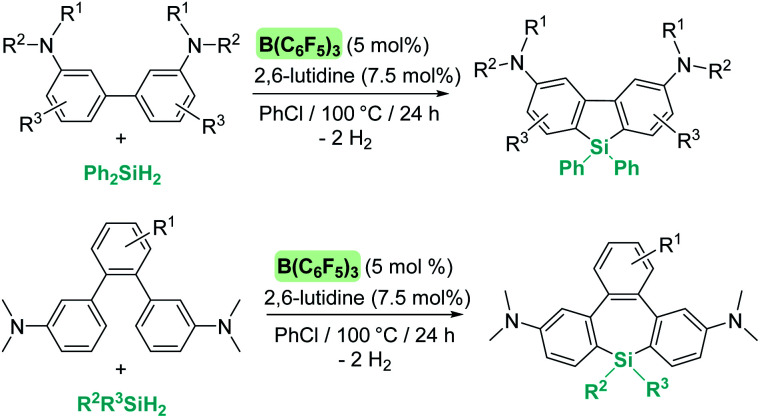
Synthesis of dibenzosiloles and tribenzosilepins.

**Scheme 53:**
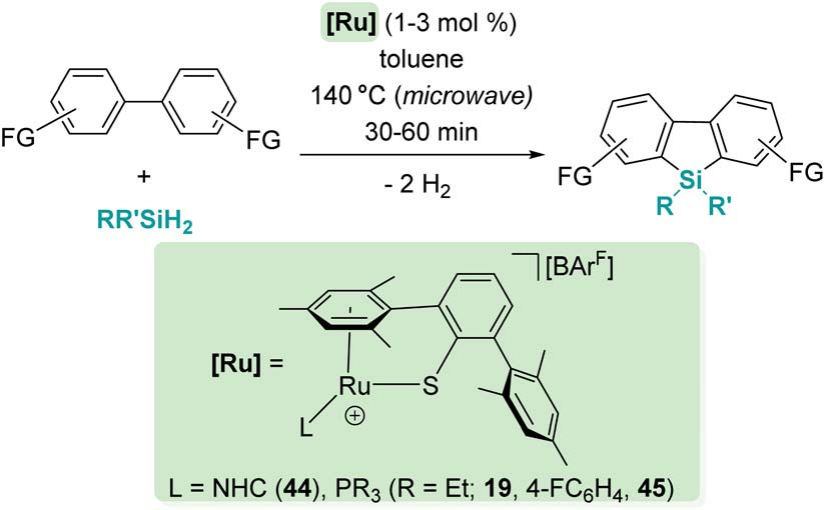
Synthesis of dibenzosiloles catalysed by ruthenium complexes.

Other electron-rich aromatic compounds can be silylated. In particular, anilines have been found to be useful reagents to be selectively silylated in their C4-position using B(C_6_F_5_)_3_ as a catalyst (Scheme 51).^106^ This catalyst tolerates primary, secondary and tertiary silanes (alkylic or aromatic) but has some tolerance problems to functional groups such as alkenes or ethers. The amines are required to be rather electron-rich, since triphenylamine is not a suitable starting material for this process. In the same vein, amino biphenyl derivatives have been used to synthesise dibenzosiloles and tribenzosilepins (Scheme 52).^107^ The synthesis of dibenzosiloles can be achieved without the assistance of the amino groups from pre-formed *ortho*-silylated biphenyls. B(C_6_F_5_)_3_ and ruthenium complexes have been demonstrated to be suitable catalysts for these transformations. With respect to the former, Ingleson reported that B(C_6_F_5_)_3_ is active in the presence of bases such as 2,6-dichloropyridine, although the scope of the reaction has not been systematically explored.^108^ Nevertheless, Oestreich proved that ruthenium complexes 19, 44 and 45 are rather active without the need for added base and tolerant to both electron-donating and electron-withdrawing groups (Scheme 53).^109^

## 4. Catalytic hydroboration reactions

Keeping in mind all the aforementioned results it comes as no surprise that some efforts have been made towards the generation of boron-based cations (borenium or boronium) from hydroboranes and their utilization in catalytic hydroboration reactions. Nevertheless, the number of reports in this area is still small, particularly with respect to transition metals, but some promising results have come to light, as described below.

**Scheme 54:**
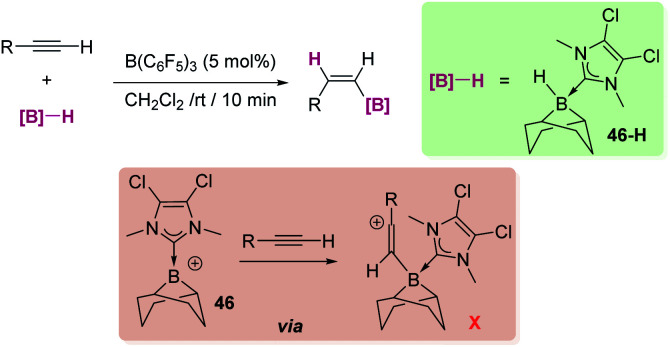
Hydroboration of alkynes catalysed by B(C_6_F_5_)_3_.

The hydroboration of alkynes and alkenes by boron-based Lewis acids has been reported. However, caution has to be taken regarding the mechanism of this transformation. Although one might be tempted to think about a mechanism involving formation of borenium/boronium cations by H-abstraction from the borane,^110^ mechanistic analyses by Stephan,^111^ Thomas, Cowley, Duarte^112^ and Oestreich^113^ indicate that the reaction mechanism can be considerably more complex. For example, they have observed a change in the nature of the catalyst due to reaction with the alkene/alkyne or through rearrangements with the borane used as a reagent, with no heterolytic cleavage of the BH bond induced by the Lewis acid. Nevertheless, in some occasions, borenium intermediates have been detected in the hydroboration of alkynes. That is the case of the report by Ingleson *et al.*, in which they have shown that B(C_6_F_5_)_3_ promotes the formation of NHC-stabilised borenium cation 46 from the NHC–BH (46-H in Scheme 54) adduct that is involved in the *trans*-hydroboration of alkynes.^114^ It is not clear whether the transfer of the hydride to the transient vinyl cation X leading to the final alkene is delivered by HB(C_6_F_5_)_3_^−^ (that would regenerate B(C_6_F_5_)_3_) or by borane 46-H (that would restore a borenium cation). A similar reaction mechanism has been proposed by Du, Feng, *et al.* for the hydroboration of alkenes by the NHC stabilised borane IMe·BH_3_ (IMe = 1,3-dimethyl-imidazole-2-ylidene), using B(C_6_F_5_)_3_ as a catalyst.^115^

**Scheme 55:**
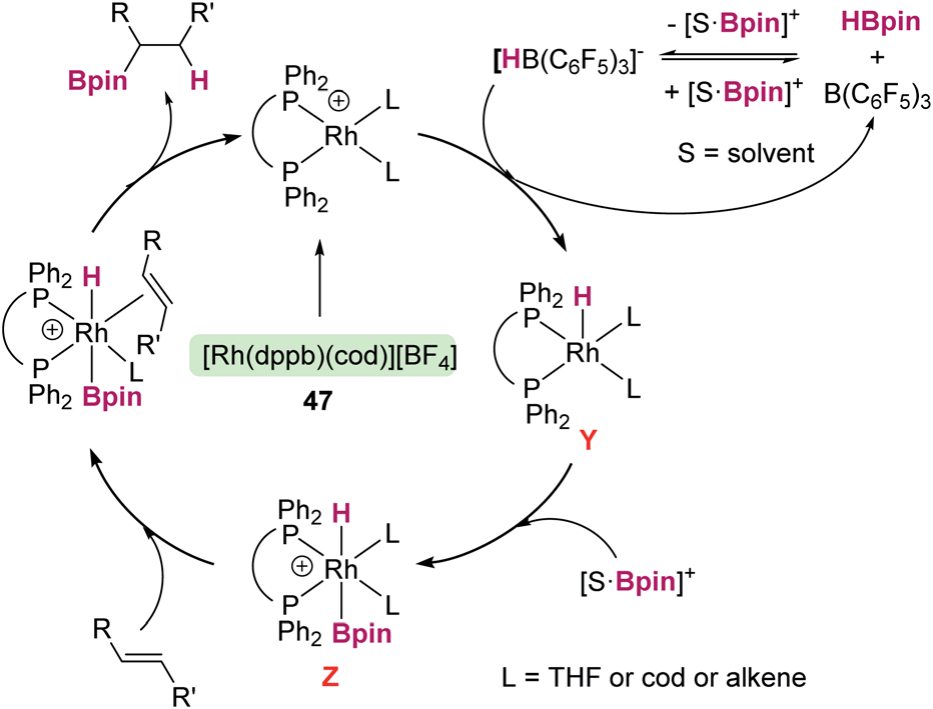
Mechanistic proposal for the hydroboration of alkenes catalysed by 47/B(C_6_F_5_)_3_.

We are not aware of catalytic processes of this type involving transition metal complexes. However, an interesting result came from the group of Crudden. They reported that hydroboration of terminal and internal alkenes can be carried out using a combination of B(C_6_F_5_)_3_ and a cationic rhodium pre-catalyst, [Rh(dppb)(cod)][BF_4_] (dppb = 1,4-bis(diphenylphosphino)butane), 47.^116^ Under certain reaction conditions (non-coordinating solvents such as dichloroethane) neither the B(C_6_F_5_)_3_ nor the cationic rhodium complex alone catalyses the hydroboration of alkenes. Nevertheless, hydroboration takes place in coordinating solvents (such as THF), but the selectivity of the reaction is highly influenced by the use of B(C_6_F_5_)_3_ as a co-catalyst when using internal alkenes (with preference towards linear hydroboration products *vs.* branched products). The authors noticed that no reaction is apparently observed when mixing B(C_6_F_5_)_3_ and HBpin, but formation of HB(C_6_F_5_)_3_^−^ is detected upon adding the rhodium catalyst, which should be accompanied by the formation of a borenium cation, THF·Bpin^+^. They came to the conclusion that a possible reaction pathway under the catalytic conditions involves initial hydride transfer from HB(C_6_F_5_)_3_^−^ to the cationic rhodium complex (Scheme 55). This would lead to a neutral rhodium hydride species Y that can trap the generated borenium cation yielding a cationic rhodium hydride boryl complex Z in which the rhodium is formally undergoing a change in its oxidation state from rhodium(i) to rhodium(iii). From here, the olefin can bind the metal centre and in subsequent processes undergo hydroboration, regenerating the initial rhodium(i) catalyst after reductive elimination. Although the whole mechanistic picture of the reaction is still unclear this is an example of the great potential of combining transition metal catalysts and p-block Lewis acids.

**Scheme 56:**
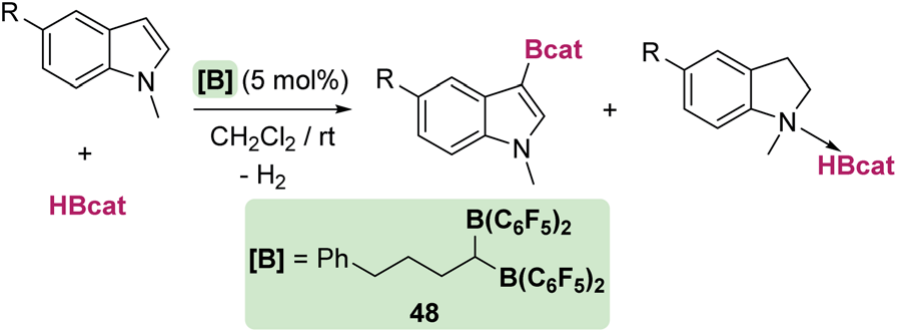
CH borylation of indoles catalysed by 48.

There are some reports regarding C–H bond borylation reactions or reduction of pyridines that point to some similarities between late transition metals and p-block based Lewis acids. For example, Erker *et al.* analysed the catalytic activity of a series of electrophilic boranes in the borylation of C–H bonds of heteroarenes and electron-rich arenes.^117^ B(C_6_F_5_)_3_ was shown to catalyse the C3 borylation of *N*-methylindole with low efficiency (35% yield of product after 1 h at room temperature), whereas the geminal bis-borane Ph(CH_2_)_3_CH[B(C_6_F_5_)_2_]_2_, 48, was noticeably more active (56% yield under the same reaction conditions) (Scheme 56). It is worth noting that the analogous monoboryl catalyst Ph(CH_2_)_3_B(C_6_F_5_)_2_ does not catalyse the reaction, highlighting the relevance of having two electrophilic boranes. More importantly, the authors suggested that the reaction mechanism follows a similar ionic pathway to that reported for C3 silylation reactions of indoles. In this case, hydride abstraction by the catalyst takes place, after which the generated borenium is trapped *via* nucleophilic attack of the C3 atom of the indole. Then, a deprotonation process yields the final products. Interestingly, indolines are also observed as side-products in the reaction, having a similar origin to the indolines generated during silylation processes. Similarly, anilines can be selectively borylated in their C4 position.

**Scheme 57:**
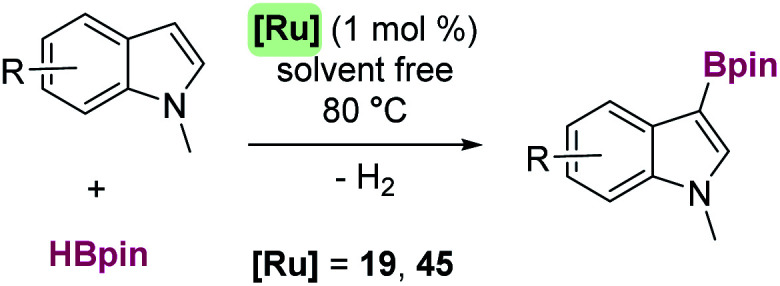
CH borylation of indoles catalysed by 19 and 45.

Ruthenium complexes have been demonstrated to be catalytically competent in the borylation of N-heterocyclic compounds (indoles and pyrroles). Oestreich, Tatsumi and co-workers observed that ruthenium complexes 19 and 45 react with different primary boranes leading to a ruthenium hydride complex bearing a sulfur-stabilised borenium cation, in which the extent of B–H bond cleavage depends on the borane.^118^ With these results in hand, they explored the catalytic activity of the ruthenium complexes in the C3 borylation of indoles and found that this is actually possible under heating at 80 °C without the need for solvent or the use of an external base (Scheme 57). At variance with the aforementioned results using boron-based Lewis acids as catalysts, the reaction does not produce indolines, yielding exclusively the C3 indole borylated products. In contrast, this catalytic system was not sufficiently reactive to borylate *N*-dimethylaniline.

## 5. Catalytic dehydrocoupling of amine-boranes

**Scheme 58:**
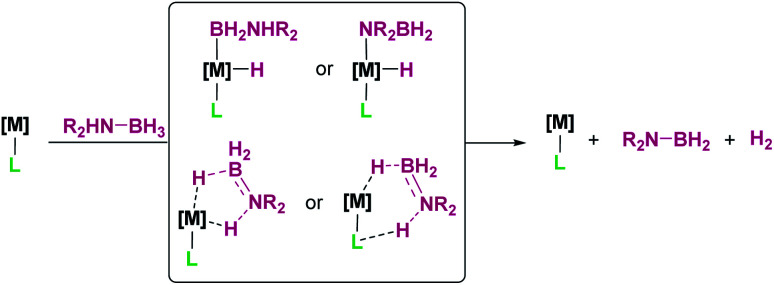
Classical mechanisms for metal-catalysed dehydrocoupling of amine-boranes.

Amine-boranes NR_2_H·BH_3_ (R = alkyl, aryl, H) have been a topic of intense research for two main reasons. On one hand, they have been considered as hydrogen storage materials, in particular ammonia borane NH_3_·BH_3_, due to its high hydrogen content (19.6 wt% H_2_). On the other hand, they have been used as precursors for the generation of polymers based on boron and nitrogen through dehydropolymerization processes.^119^ Although they can release dihydrogen under uncatalysed conditions at high temperatures, H_2_ extrusion can be more appropriately induced by metal and non-metal catalysed processes. The main advantage is the lowering of the temperatures necessary to promote the dehydrocoupling, offering some control over the properties of the polymeric materials formed through boron–nitrogen coupling reactions. From a mechanistic point of view, metal-catalysed dehydrocoupling processes have been shown to be diverse and might involve the initial activation of the B–H or N–H bond, or both of them in a concerted fashion, either by the metal itself or assisted by some of the ligands coordinated to the metal centre (Scheme 58).^120^

### 5.1 Dehydrocoupling of amine-boranes catalysed by boron-based Lewis acids

**Scheme 59:**
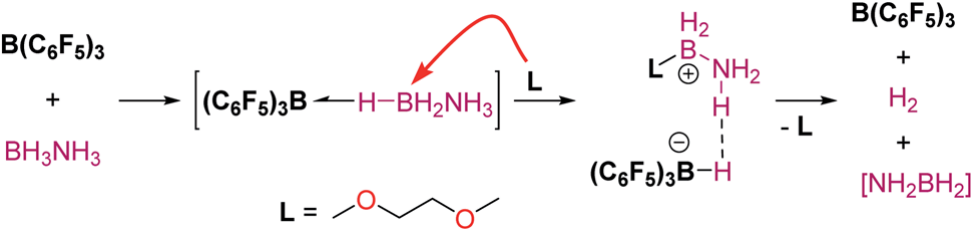
Mechanistic proposal for the dehydrocoupling of NH_3_·BH_3_.

The activation modes of the aforementioned amine-boranes are not suitable, *a priori*, for p-block Lewis acids, or might not be possible for some electrophilic transition metals. Nevertheless, an alternative mechanism involving hydride abstraction by the Lewis acid with concomitant formation of borenium or boronium ions^121^ could trigger a dehydrogenation process. Baker, Dixon, *et al.* reported^122^ that upon mixing B(C_6_F_5_)_3_ (25 mol%) and ammonia borane, 0.6 equiv. of H_2_ are produced at 60 °C in diglyme together with the ion pair [HB(C_6_F_5_)_3_][BH_2_(NH_3_)]. The latter arises from hydride abstraction from H_3_B·NH_3_ by B(C_6_F_5_)_3_, which generates the borenium cation (NH_3_)BH_2_^+^ (likely in the form of the boronium cation (NH_3_)BH_2_(L)^+^; L = solvent). Cyclic products containing B–N units ([H_2_NBH_2_]_*n*_) were also detected by ^11^B NMR spectroscopy. The mechanism of this process has been recently addressed by Paul *et al.*^24*a*^ According to their calculations, the process starts with formation of a σ-BH complex (Scheme 59). Similar to the silane chemistry described before, the boron atom of H_3_B·NH_3_ is prone to nucleophilic attack by a solvent molecule (glyme in the calculations), facilitating the transfer of a hydride to B(C_6_F_5_)_3_. This process, leading to [HB(C_6_F_5_)_3_]^−^ and boronium cation [H_2_B(NH_3_)(glyme)]^+^, requires overcoming an energy barrier of 10.9 kcal mol^−1^. Then, the acidic NH protons of the boronium cation can react with [HB(C_6_F_5_)_3_]^−^ releasing H_2_ at an energy cost of 33.8 kcal mol^−1^. This last step regenerates B(C_6_F_5_)_3_ and releases H_2_BNH_2_, which is unstable and generates polycyclic oligomers and polymers.

**Scheme 60:**
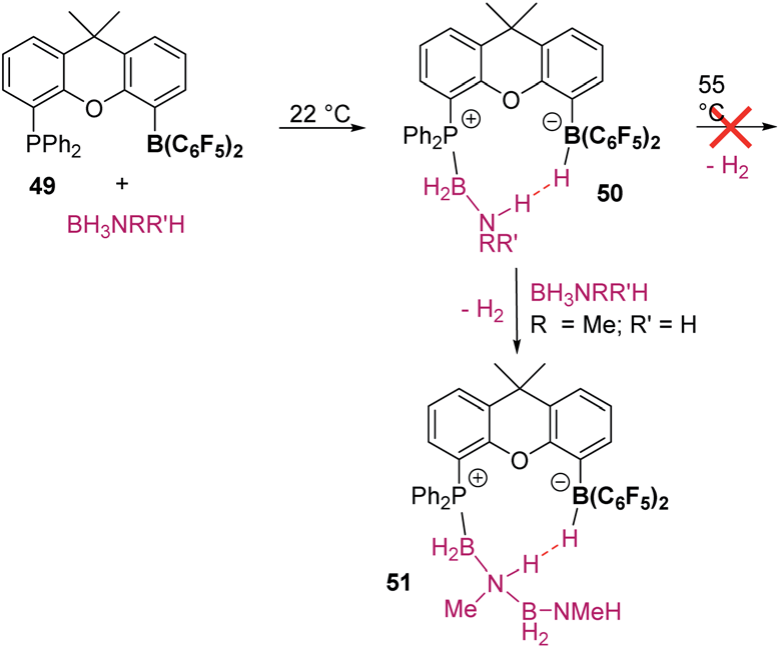
Dehydrocoupling of amine boranes mediated by 49.

On the other hand, Miller *et al.* found that B(C_6_F_5_)_3_, in combination with P^*t*^Bu_3_ (a well-known combination as a frustrated Lewis pair), dehydrogenates amine boranes NR_2_H·BH_3_ (R = Me, H) at 25 °C in chlorobenzene releasing 1 equiv. of H_2_, leading to the ion pair [P^*t*^Bu_3_H][HB(C_6_F_5_)_3_] and [NR_2_BH_2_]_*x*_ (*x* = 2 for R = Me and *x* = *n* for R = H).^123^ The authors suggested that the process involves the initial abstraction of a hydride from the amine-borane, followed by deprotonation of the acidic NH proton of the generated boronium cation by P^*t*^Bu_3_, thus explaining the formation of [P^*t*^Bu_3_H][HB(C_6_F_5_)_3_]. Nevertheless, the catalytic process is hampered by the stability of [P^*t*^Bu_3_H][HB(C_6_F_5_)_3_] towards H_2_ extrusion, which requires heating to at least 150 °C. In this regard, Aldridge and co-workers envisioned that a dimethylxanthene FLP 49 (Scheme 60) based on boron and phosphorus would attenuate considerably this drawback, since H_2_ extrusion is rather favourable for this system.^124^ This FLP 49 (1 mol%) was, in fact, able to dehydrogenate amine-boranes (including ammonia borane) at 55 °C. Curiously, the mechanism for H_2_ formation involves a formal deprotonation of the NH of a phosphine-stabilised boronium cation by another molecule of amine-borane. Stoichiometric reactions between amine-boranes and 49 resulted in the formation of the phosphine-stabilised boronium cation 50. In spite of the favourable orientation of the acidic NH proton and the hydride of [HB(C_6_F_5_)_3_]^−^ for hydrogen extrusion, the molecule is stable up to at least 55 °C. Paul *et al.* have calculated a high energy barrier of 40.8 kcal mol^−1^ to release H_2_ from 50.^125^ However, if another molecule of amine-borane is added to this boronium cation, hydrogen is produced together with a new species 51, arising from a boron–nitrogen bond coupling between the terminal NH fragment and the BH of the added amine-borane. Addition of a third equivalent of amine-borane is responsible for the release of cyclic trimers ([H_2_BNRR′]_3_, borazines, *etc.*), although the mechanism for this last transformation is not entirely clear.

### 5.2 Dehydrocoupling of amine-boranes catalysed by transition metal-based Lewis acids

**Scheme 61:**
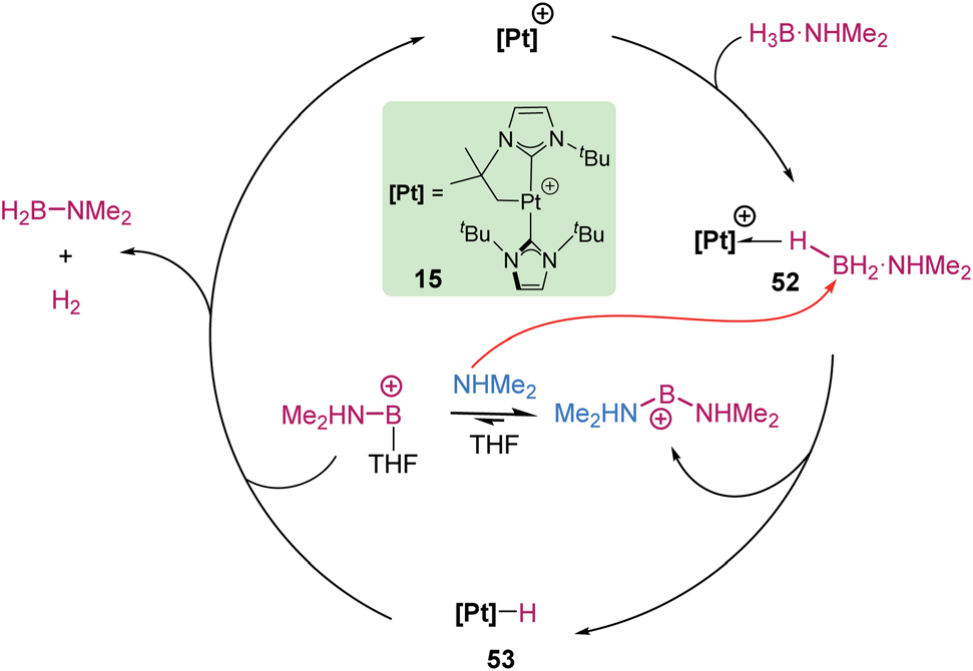
Mechanism for the dehydrocoupling of NMe_2_H·BH_3_ catalysed by 15.

Our group reported in 2013 that the low electron count platinum(ii) complex [Pt(I^*t*^Bu′)(I^*t*^Bu)][BAr^F^] (15) is able to dehydrogenate NMe_2_H·BH_3_ at room temperature, leading to the amino-borane [NMe_2_BH_2_]_2_ and H_2_.^126^ Low temperature NMR studies allowed us to detect some relevant intermediates involved in the reaction mechanism (Scheme 61). First, the Shimoi-type σ-BH complex [Pt(I^*t*^Bu′)(I^*t*^Bu)(η^1^-HBH_2_·NMe_2_H)][BAr^F^], 52, was observed at temperatures below 0 °C and calculated (by DFT methods) to be more stable than the starting complex. As the temperature increases, hydrogen atom transfer from boron to platinum takes place assisted by a molecule of free NMe_2_H (arising from dissociation from amine-borane NMe_2_H·BH_3_), producing the neutral platinum hydride complex [Pt(H)(I^*t*^Bu′)(I^*t*^Bu)], 53, and the boronium cation [(BH_2_)(NHMe_2_)_2_][BAr^F^]. The latter is finally involved in the protonation of the platinum hydride 53. Therefore, the behaviour of this platinum complex was alike that reported for B(C_6_F_5_)_3_, with no change in the oxidation state at the metal throughout the process. However, the rate limiting step of the reaction with platinum is predicted to be (according to DFT calculations) the B–H bond splitting (24.3 kcal mol^−1^; 10.9 kcal mol^−1^ for B(C_6_F_5_)_3_), instead of the protonation event.

**Scheme 62:**
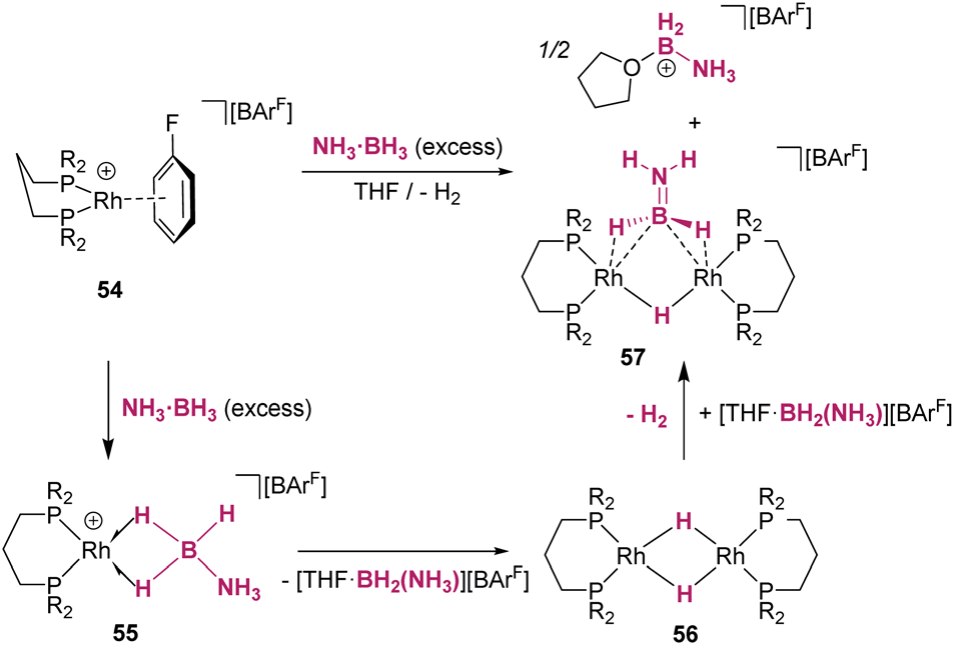
Dehydrocoupling of NH_3_·BH_3_ mediated by 55.

Boronium cations have been detected as intermediates in dehydrocoupling processes of amine boranes using rhodium complexes. Weller *et al.* reported that stoichiometric addition of NH_3_·BH_3_ to the cationic complex [Rh(dppp)(η^6^-C_6_H_5_F)][BAr^F^], 54, resulted in the generation of the binuclear complex 57 (Scheme 62), with a coordinated amino-borane NH_2_–BH_2_, and the boronium cation [THF·BH_2_(NH_3_)][BAr^F^].^127^ As in the case described before for platinum, the mechanism involves the initial formation of a bis-η^1^-BH complex 55 (spectroscopically detected for R = ^i^Pr), followed by hydride transfer from boron to rhodium. This last step is responsible for the formation of a dinuclear rhodium hydride complex 56 and the boronium cation [THF·BH_2_(NH_3_)][BAr^F^]. Reaction of one of the hydrides of the rhodium complex with the acidic NH proton yields complex 57, H_2_ and half equivalent of unreacted [THF·BH_2_(NH_3_)][BAr^F^]. The dehydrogenation of ammonia borane can be carried out catalytically using 0.5 mol% of the rhodium complex at room temperature in THF (3 h). Under these conditions, 1.2 equivalents of H_2_ are produced together with oligomeric species (among which B-(cyclotriborazanyl)amine-borane was detected) and polyaminoborane. Other amine-boranes such as NMeH_2_·BH_3_ undergo a dehydropolymerization process leading to 1.1 equivalents of H_2_ and polymethylamino-borane [H_2_BNMeH]_*n*_.

**Fig. 11:**
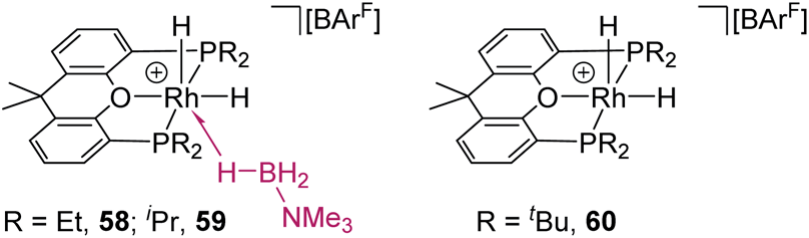
Rhodium complexes used in dehydropolymerization processes.

Rhodium complexes [Rh(κ^3^-P,O,P-xantphos-R)(H)_2_(η^1^-HBH_2_·NMe_3_)][BAr^F^] (R = Et, 58; ^i^Pr, 59) and [Rh(κ^3^-P,O,P-xantphos-^*t*^Bu)(H)_2_][BAr^F^], 60 (Fig. 11) are also catalytically active in the dehydropolymerization of amine-borane NMeH_2_·BH_3_ using as low as 0.2 mol% of catalyst at room temperature.^128^ The best catalyst appeared to be complex [Rh(κ^3^-P,O,P-xantphos-^i^Pr)(H)_2_(η^1^-HBH_2_·NMe_3_)][BAr^F^], bearing an isopropyl-substituted xantphos phosphine (conversions up to 98% in 20 min leading to polyaminoborane [H_2_BNMeH]_*n*_ of low molecular weight and high dispersity (*M*_*n*_ = 9000 g mol^−1^, *Đ* = 2.9)). Although the mechanistic picture of the process is rather complex, the authors suggested the following: first, substitution of NMe_3_·BH_3_ by NMeH_2_·BH_3_ (in the case of 58 and 59) yields intermediate [Rh(κ^3^-P,O,P-xantphos-R)(H)_2_(η^1^-HBH_2_·NMeH_2_)][BAr^F^]. Then, hydride transfer from boron to rhodium takes place with assistance from free NMeH_2_, giving neutral rhodium(iii) hydride [Rh(κ^3^-P,O,P-xantphos-R)(H)_3_] and the boronium cation [(NH_2_Me)_2_BH_2_][BAr^F^]. Subsequently, deprotonation of the latter in the presence of NMeH_2_·BH_3_ regenerates [Rh(κ^3^-P,O,P-xantphos-R)(H)_2_(η^1^-HBH_2_·NMeH_2_)][BAr^F^]. Nevertheless, alternative reaction pathways involving intramolecular N–H bond activation by rhodium hydride species were not ruled out.

**Scheme 63:**
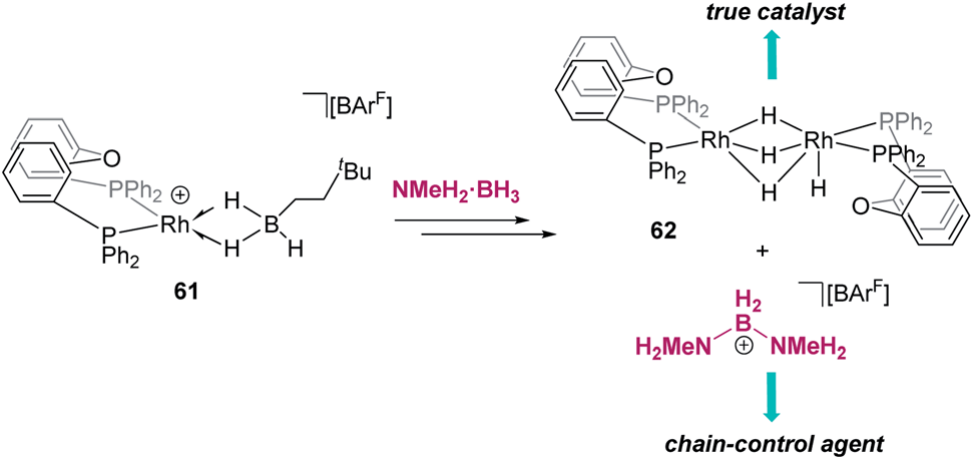
Dehydropolymerization of NMeH_2_·BH_3_ mediated by 61.

In a series of more recent contributions, Weller and co-workers showed that in spite of the ability of rhodium complex [Rh(DPEphos)(η^2^-H_2_BNMe_3_(CH_2_)_2_^*t*^Bu)][BAr^F^] (61) (Scheme 63) to generate boronium cations and rhodium hydride species from amine-boranes, they are not involved in the deprotonation of the NH fragment, contrary to the results described previously.^129^ According to their exhaustive and complex study, they concluded that the generated dinuclear rhodium hydride 62 is the true active species, whereas the boronium cation plays a role in controlling the degree of amine-borane polymerization (chain-control agent).

**Scheme 64:**
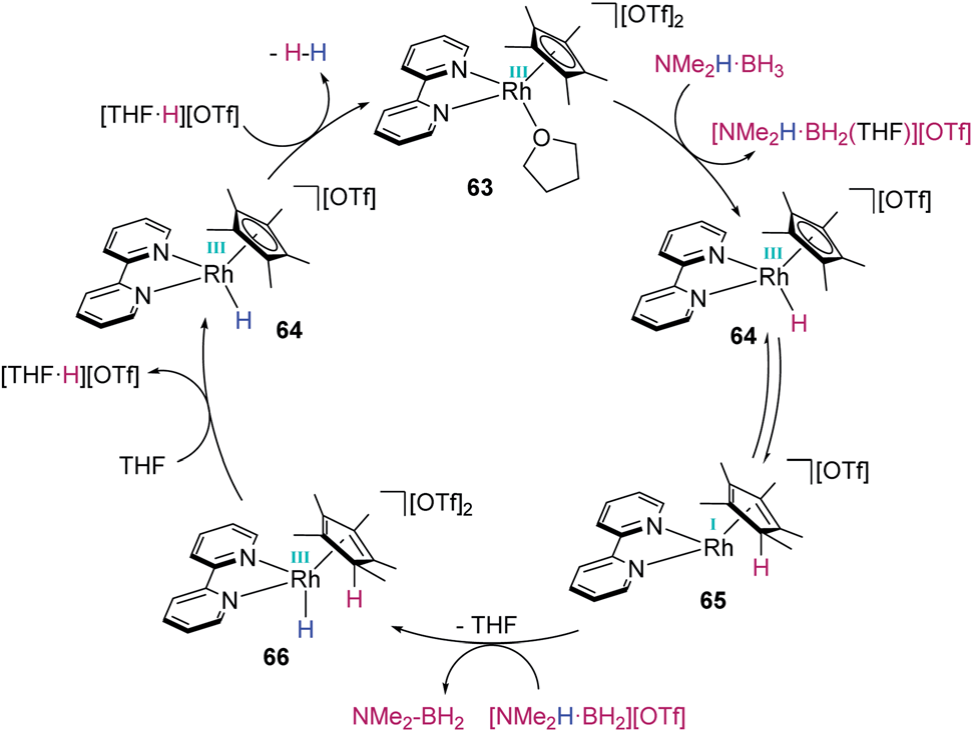
Dehydrocoupling of NMe_2_H·BH_3_ catalysed by 63.

In some occasions, metal–ligand cooperativity has been shown to mediate dehydrocoupling processes. This is the case of the cyclopentadienyl ligand in the dicationic rhodium complex [Cp*Rh(bipy)(THF)][OTf]_2_, 63, reported by Nozaki *et al.*^130^ This compound is able to dehydrogenate NMe_2_H·BH_3_ into amino-borane [NMe_2_–BH_2_]_2_ at 50 °C in THF using only 0.025 mol% of the catalyst (Scheme 64). In this process, hydride abstraction by the rhodium complex initiates the catalysis, resulting in the expected THF stabilised borenium cation [BH_2_·(NMe_2_H)(THF)][OTf] together with a cationic rhodium hydride complex [Cp*Rh(bipy)(H)][OTf], 64. However, besides this latter complex, another isomeric species was detected by NMR spectroscopy that was identified as complex 65 in Scheme 64, formally arising from migration of the hydride from rhodium to the Cp* ligand. This complex is in equilibrium with the hydride derivative 64. DFT calculations indicated that direct protonation of the rhodium hydride 64 by the N–H bond of [BH_2_·(NMe_2_H)(THF)][OTf] is actually higher in energy than an alternative reaction pathway involving protonation of the rhodium centre in complex 65 (with η^4^-Cp*) leading to intermediate 66. This intermediate could, in principle, undergo a concerted H_2_ extrusion, but the authors found that this step is energetically prohibitive (43.5 kcal mol^−1^). However, deprotonation of the acidic C–H bond of the Cp* ligand by a weak base such as THF is energetically viable (2.0 kcal mol^−1^) and protonation of the regenerated rhodium hydride by THF·H^+^ is almost barrierless (0.2 kcal mol^−1^). This last step regenerates the catalyst and forms H_2_ in an overall process that is exergonic by 2.1 kcal mol^−1^. Interestingly, the iridium analogue [Cp*Ir(bipy)(THF)][OTf]_2_ exhibited a poor catalytic activity. This was attributed, in part, to the difficulty of transferring the hydride from iridium to the Cp* ligand, a process that, at variance with rhodium, was not observed. Therefore, the Cp* ligand is cooperating in the process and THF acts as a proton shuttle. Importantly, and contrary to the previous catalysts described here, the rhodium centre needs to undergo a formal redox process from rhodium(iii) to rhodium(i) and again rhodium(iii).

**Scheme 65:**
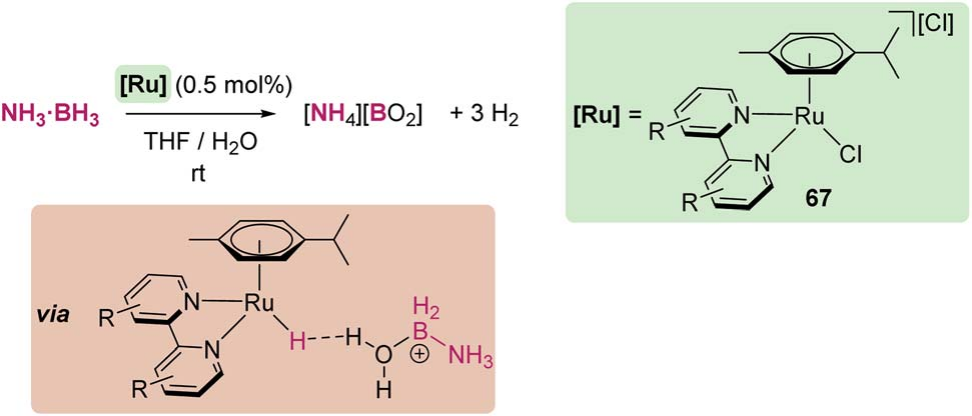
Hydrolysis of NH_3_·BH_3_ catalysed by 67.

Amine-boranes, and particularly ammonia borane, have been the subject of solvolysis processes for hydrogen storage. Recently, Freixa *et al.* analysed the mechanism by which ammonia borane undergoes B–H/O–H dehydrocoupling by ruthenium complexes [Ru(*p*-cym)(bipy^R^)Cl][Cl], 67 (were *p*-cym is *para*-cymene and bipy^R^ a substituted bipyridine), producing up to 3 equivalents of H_2_ and B–O by-products (Scheme 65).^131^ Through a series of NMR and mass-spectrometry experiments, the authors were able to detect boronium cations and ruthenium hydrides as intermediates. In this case, the OH fragment of the generated boronium cation [BH_2_(NH_3_)(ROH)]^+^ (with R = H or alkyl group) is deprotonated by the ruthenium hydride complex. It was observed that the best catalyst is that bearing electron-withdrawing groups at the bipy^R^ ligand (such as NO_2_).

In summary, dehydrocoupling reactions of amine-boranes catalysed by boron-based Lewis acids and electrophilic transition metals seem to follow very similar pathways. In most of the cases, this involves initial hydride abstraction by the Lewis acid (with potential assistance by an external Lewis base) with concomitant formation of boronium/borenium cations. The latter contain acidic NH or OH protons that react with the hydride–Lewis acid species, releasing dihydrogen and the dehydrocoupled borane. Nevertheless, extrusion of H_2_ might proceed *via* alternative reaction pathways involving the participation of another molecule of borane (as in Scheme 60) or a metal centre (as in Scheme 64).

## Conclusions

σ-EH (E = Si, B) transition metal complexes can polarise EH bonds, making the E atom susceptible to nucleophilic attack by an appropriate nucleophile, before any possible oxidative addition process of the E–H bond takes place. Construction of E–C, E–N, and E–O bonds as well as defunctionalisation processes breaking C–O bonds have been possible under catalytic conditions with excellent activities. Thus, their reactivity parallels that observed for main group-based Lewis acids (mostly boron and aluminium), and involves the initial formation of transient silylium and borenium/boronium cations and metal hydride species. Still, some of the subsequent steps might follow different reaction pathways, as a consequence of the more flexible reactivity of transition metals and the potential participation of the ligands around them. The number of processes and metal centres undergoing this type of reactivity has increased considerably in recent years and in some cases the activity of the catalyst for a specific reaction can be even higher than that observed for main group-based catalysts. In this context, B(C_6_F_5_)_3_ is still the most popular and versatile catalyst and some catalytic processes have not yet been developed with transition metal-based catalysts. However, although the modulation of the electronic and steric properties of boron-based Lewis acids has been addressed, the combination of transition metals and different ligand environments (including the possibility of metal–ligand cooperativity) could provide a more versatile tool for controlling the reactivity of σ-EH complexes. In addition, it is not entirely understood to what extent an η^1^ coordination mode may be beneficial in comparison to η^2^, and more intense research should be done to increase the number of transition metal complexes favouring η^1^ σ-EH type binding. In our group, we have reported that some electron-deficient platinum(ii) complexes are able to facilitate this end-on coordination mode for both silanes and boranes (particularly with tricoordinated boranes), although their reactivity is yet to be explored. Moreover, the future of electrophilic activation of Si–H and B–H bonds would likely hinge on the use of first row metals instead of precious metals. We expect that novel catalytic transformations are to be discovered using these principles.

## Author contributions

All authors were involved in the preparation of the manuscript. The final manuscript was approved by all authors.

## Conflicts of interest

There are no conflicts to declare.

## Supplementary Material
